# Comparative Ecology of *Bartonella* and *Brucella* Infections in Wild Carnivores

**DOI:** 10.3389/fvets.2018.00322

**Published:** 2019-01-04

**Authors:** Michael Kosoy, Irina Goodrich

**Affiliations:** Centers for Disease Control and Prevention, Fort Collins, CO, United States

**Keywords:** *Bartonella*, *Brucella*, carnivores, disease ecology, wildlife disease

## Abstract

Phylogenetic sister clades *Bartonella* and *Brucella* within the order Rhizobiales present some common biological characteristics as well as evident differences in adaptations to their mammalian reservoirs. We reviewed published data on *Bartonella* and *Brucella* infections in wild carnivores to compare the ecology of these bacteria in relatively similar host environments. Arthropod vectors are the main mechanism for *Bartonella* species transmission between mammalian hosts. The role of arthropods in transmission of *Brucella* remains disputed, however experimental studies and reported detection of *Brucella* in arthropods indicate potential vector transmission. More commonly, transmission of *Brucella* occurs via contact exposure to infected animals or the environment contaminated with their discharges. Of 26 species of carnivores tested for both *Bartonella* and *Brucella*, 58% harbored either. Among them were bobcats, African lions, golden jackals, coyotes, wolves, foxes, striped skunks, sea otters, raccoons, and harbor seals. The most common species of *Bartonella* in wild carnivores was *B. henselae*, found in 23 species, followed by *B. rochalimae* in 12, *B. clarridgeiae* in ten, and *B. vinsonii* subsp. *berkhoffii* in seven. Among *Brucella* species, *Br. abortus* was reported in over 30 terrestrial carnivore species, followed by *Br. canis* in seven. Marine carnivores, such as seals and sea lions, can host *Br. pinnipedialis*. In contrast, there is no evidence of a *Bartonella* strain specific for marine mammals. *Bartonella* species are present practically in every sampled species of wild felids, but of 14 *Brucella* studies of felids, only five reported *Brucella* and those were limited to detection of antibodies. We found no reports of *Bartonella* in bears while *Brucella* was detected in these animals. There is evident host-specificity of *Bartonella* species in wild carnivores (e.g., *B. henselae* in felids and *B. vinsonii* subsp. *berkhoffii* in canids). A co-adaptation of *Brucella* with terrestrial wild carnivore hosts is not as straightforward as in domestic animals. Wild carnivores often carry the same pathogens as their domesticated relatives (cats and dogs), but the risk of exposure varies widely because of differences in biology, distribution, and historical interactions.

## Introduction

Sixty percent of emerging infectious diseases are zoonoses and majority of these (71.8%) originate from wildlife (1). Among pathogens, *Bartonella* species might represent an underappreciated danger for human and animal health (2) and human brucellosis remains one of the most common zoonotic diseases worldwide, with more than 500,000 new cases every year (3).

*Bartonella* and *Brucella* are phylogenetic sister clades in the order Rhizobiales (4, 5). The genus *Brucella* is composed of 12 recognized species defined to their preferential hosts (6, 7). The more diverse genus *Bartonella* includes over 33 validated species exhibiting extremely high genetic diversity (8). Genome analyses of representative species of these bacterial genera have confirmed their shared ancestry. Alsmark et al. (9) identified 760 *Bartonella henselae* genes, for which homologs are present in one of chromosomes of *Brucella suis*. In addition to their genetic proximity, the *Bartonella* and *Brucella* genera present analogies in their life history and ecology that are even more important for our analysis. Whereas, most closely related species of the order Rhizobiales are symbiotic on plant roots, both *Bartonella* and *Brucella* are adapted to diverse mammalian hosts. Each *Bartonella* and *Brucella* species has one or a few closely related mammal reservoir hosts (5).

Investigations of wild animals, including predators, for *Brucella* infections started much earlier and were more intensive compared to studies of *Bartonella* infections. Research of *Brucella* infections in animals has been dominated by studies of domestic animals and, to a lesser degree, of wild ruminants. Although *Brucella canis* was identified in domestic dogs more than 50 years ago and is well known to veterinary community as a causative agent of canine abortion (10), investigations of *Brucella* in dogs are much fewer than those of *Brucella* in cattle, sheep, goats, and pigs. This is mainly due to lack of good tests for rough *Brucella* species, not because of lack of interest. Publications on the distribution of *Brucella* species among wild canids, as well as among other wild carnivores, are even more limited in the western literature. At the same time, a good number of reports on this topic are scattered across Russian literature. Most of these were published during Soviet times, sometimes in classified proceedings, and are not easily available (11–13). Identification of novel species and genotypes of *Brucella* in rodents, bats, marine mammals, and amphibians stimulated epidemiological research of diverse animal species, including wildlife (7).

Extensive investigations of animals for *Bartonella* species started in the early 1990s after the discovery that one or more *Bartonella* species could cause cat scratch disease in people. For this reason, most studies targeted domestic cats and dogs with limited investigations of stray dogs and feral cats (14, 15). Studies on detection, identification, and characterization of *Bartonella* species in wild animals usually targeted small mammals: rodents (8) and bats (16). Chomel et al. (17) pioneered *Bartonella* research in wild carnivores and ruminants. Since then, numerous wildlife studies have been conducted in various parts of the world. However, a comprehensive analysis of the available data on prevalence and diversity of *Bartonella* and *Brucella* infections in wild carnivores has yet to be published. Such an analysis would allow comparisons to be made between the ecologies of these two bacterial groups living in similar host environments and identify possible directions for future research.

In this review we undertook such an analysis through an extensive literature review. We examined the similarities and differences in the *Bartonella* and *Brucella* ecology and, more importantly, analyzed biological features that may reveal ways of these phylogenetically close bacterial genera exhibit evolutionary adaptations to the same or related mammalian hosts, presumably during the long periods over which they have co-occurred. Considering the differences in the genera's life history, we paid special attention to possible arthropod-mediated transmission of these bacteria between mammalian hosts.

For this review, we followed the more accepted taxonomic division of the order Carnivora into suborders Feliformia (“cat-like”) and Caniformia (“dog-like”), with pinnipeds included as a separate superfamily level clade (Pinnipedia). We chose these divisions not for preference for a specific taxonomic scheme, but as a convenient basis for analysis of available data on *Bartonella* and *Brucella* infections in 12 families: suborder Feliformia (Felidae, Herpestidae, Hyaenidae, and Viverridae), suborder Caniformia (Canidae, Mephitidae, Mustelidae, Procyonidae, and Ursidae), and clade Pinnipedia (Odobenidae, Otariidae, and Phocidae).

We conducted a thorough literature search by using PubMed, Scopus, OVID Medline, BioOne, Crossref, WorldCat, Web of Science, Google Scholar, and other databases. In the search we used keywords: “*Bartonella* ecology,” “*Brucella* ecology,” “*Bartonella* AND wild animals,” “*Brucella* AND wild animals,” “*Bartonella* AND carnivores,” “*Brucella* AND carnivores,” “*Bartonella* AND predators,” “*Brucella* AND predators,” “*Bartonella* AND marine mammals,” “*Brucella* AND marine mammals,” “Bacteria AND wild carnivores,” “*Bartonella* AND fleas AND mammals,” “*Brucella* AND arthropods,” and their variations. We realized that all these search engines had missed numerous reports on detection of *Brucella* in wild animals in the Russian language literature and we conducted our own search of such sources in the Russian Internet and search engines as well as by working through the references in related articles and reviews in the Russian language.

We used the word “wild” in the meaning of “free-ranging” and apart from a few publications of particular interest, we excluded reports of *Bartonella* and *Brucella* in captive and zoo animals as the composition of bacterial communities in such animals could have been modified by separation from the natural environment or via acquisition of bacterial infections from the surrounding environment (e.g., from urban rats). The literature on *Bartonella* and *Brucella* infections in domestic carnivores (cats and dogs) is abundant, so we limited inclusion for comparative purposes only.

We collected data from serological, bacteriological, and molecular investigations of *Bartonella* and *Brucella* infections in all families of wild terrestrial and marine carnivores worldwide. Providing data from various techniques, we need to acknowledge that discrimination power of characterization of pure cultures and sequence analyses for identification of *Bartonella* and *Brucella* species is greater than that of serological procedures. However, serological methods remain an important tool in detection and identification of these infections in animals and should be taken in consideration with full awareness of their limitations.

Then we collated the obtained information in *Bartonella, Brucella* and combined tables by carnivore species divided into their respective families listed in alphabetical order of their Latin names. We listed information on location where the samples were collected, investigation method, prevalence and bacterial species, and reference to the study. Both positive and negative results of investigations were included. The combined table shows only references listed by carnivore species in their respective families.

## Features of *Bartonella* and *Brucella* Bacteria Related to Their Ecology in Wild Animals

### Biological Characteristics

*Bartonella* and *Brucella* bacteria share some biological characteristics, yet there are evident differences in their adaptations to their animal reservoirs. Infections caused by bacteria of both taxonomic groups can lead to a long-lasting bacteremia with ability to invade specific mammalian cells and survive inside them. Via analogous mechanisms, the specialized secretion system (T4SS) works as a molecular syringe to inject effector molecules into their target cells (18, 19). *Bartonella* and *Brucella* modulate their gene expression to adapt to the different environments during the infectious process (20, 21). The VirB systems of *Bartonella* and *Brucella* are associated with distinct groups of effector proteins that collectively mediate interactions with host cells (19).

*Bartonella* bacteria infect endothelial cells and seed into the bloodstream, colonizing erythrocytes which provide a persistence niche for the bacteria. The ability of these bacteria to exploit their reservoir hosts with diminished morbidity and to cause a high level of bacteremia justifies the definition of “elegant hemotrophic parasites” given by Birtles (22) to bartonellae. In incidental hosts, *Bartonella* infections can cause various clinical manifestations commonly without high-level bacteremias (21). In contrast to *Bartonella, Brucella* bacteria invade and multiply within mammalian host's macrophages and placental trophoblasts (18, 19, 23). Although bacteremia is common during brucellosis, data on duration and mechanisms of *Brucella* persistence in animal blood are limited.

### Transmission of *Bartonella* and *Brucella* Bacteria

The persistence of *Bartonella* bacteria in red blood cells optimizes transmission of these bacteria by blood-sucking arthropods. High prevalence and long-term bacteremia in reservoir mammals and adaptation to specific vectors seem to be the common strategy of bartonellae for transmission and host diversity (24). Many described *Bartonella* species are vector-borne bacteria transmitted by fleas, sand flies, lice, and biting flies depending on the bacteria species involved and their vertebrate reservoirs (24, 25). Experimental studies demonstrated louse and flea transmission of *B. henselae* and *B. quintana* (26–28). Some investigators provided evidence of potential role of ticks and mites in transmission of *Bartonella* species, but debates continue on their role of as vectors (25, 29). A 2008 study by Cotté et al. (30) showed that *Ixodes* spp. ticks are capable of transmitting *B. henselae* via salivary contents, but Telford and Wormser (31) found no convincing evidence that ticks were vectors of *Bartonella* species. Molecular detection of *Bartonella* spp. in terrestrial leeches (*Haemadipsa rjukjuana*) by Kang et al. (32) opens up a discussion of the pathogen transmission by land leeches.

Transcutaneous transmission of *Bartonella* via animal bites and scratches during hunting, as well as through butchering or handling wild meat is another possibility (33). Cat scratch disease, caused by *B. henselae* is the best-documented example of direct animal-to-human transmission of a *Bartonella* species by scratch or bite inoculation (14). Finkelstein et al. (27) showed that *B. henselae* can remain viable in flea feces for over 72 hours. Therefore, transmission potentially can occur via inoculation of *B. henselae* from infected flea feces into the skin via open wounds. Suspected *Bartonella alsatica* transmission from wild rabbits to humans, presumably occurring during hunting and butchering, was reported in patients with endocarditis or lymphadenitis in France (34, 35). Suspected dog bite transmission of *B. vinsonii* to a human was reported based upon serological evidence (36). There is little information on possible vertical transmission of bartonellae in animals. However, *Bartonella* species were isolated from the embryos and neonates of naturally infected cotton rats (*Sigmodon hispidus*) and white-footed mice (*Peromyscus leucopus*) (37). Experimental inoculation of *B. henselae* to adult female cats was accompanied by decreased conception or failure to maintain pregnancy (38). Considering the extensive animal reservoirs and the large number of insects that have been implicated in the transmission of *Bartonella* species, animal exposure to these organisms may be more substantial than is currently believed.

Transmission of *Brucella* occurs mainly via close contact with placenta, aborted fetuses, fetal fluids, reproductive tract discharges, and secretions (7). Infected dogs intermittently shed low concentrations of bacteria in seminal fluids and nonestrus vaginal secretions. Postabortion vaginal fluids contain a high level of bacteria and are a source of infection for other dogs (39). In addition, dogs can shed the bacteria in the saliva, nasal secretions, and urine (40). Studies suggest that the concentration of *Br. canis* in urine is higher in male than female dogs; this difference is attributed to urine contamination with seminal fluid (41). However, the role of urine as a source of infection is not fully understood (39).

There is a widely accepted perception that absence of transmission of *Brucella* species via arthropod vectors is the most essential difference in ecology of these bacteria compared to the *Bartonella* species. We found a number of rarely cited publications on either detection of *Brucella* species in arthropods or experimental studies designed to verify a possibility of vector transmission of these bacteria. Although detection of *Brucella* in arthropods collected from different sources does not often directly relate to carnivores, such information can help interpret potential mechanisms of bacterial transmission. The invasion of *Brucella* into erythrocytes and its persistence in blood suggest a possibility for transmission by bloodsucking arthropods in nature (42). Although *Brucella* may be found in erythrocytes, these bacteria exhibit strong tissue tropism and replicate within vacuoles in macrophages, dendritic cells, and placental trophoblasts. Evidence that *Brucella* species can be spread among animals by arthropods is very limited. Some Russian authors argued that parasitic arthropods, especially ticks, could preserve *Brucella* in nature and transmit them within a population from one animal to another (43, 44). Rementsova (43) listed 20 observations of *Brucella* detection in ticks. Experiments in Russia reported that both ixodid and argasid ticks were infected with *Brucella* at different phases of their development and could transmit the pathogen to uninfected animals during bloodsucking (43). *Brucella* in ticks retained their virulence even after 2 years (43). More recently, Neglia et al. (45) detected *Br. abortus* DNA and RNA in different stages of development of the sucking louse (*Haematopinus tuberculatus*).

Alimentary transmission is important for *Brucella* as proved by experimental studies in wild carnivores. Scanlan et al. (46) infected gray foxes with *Br. abortus* in dog food. Seven of eight foxes became seropositive. Neiland and Miller (47) infected six beagle dogs, two wolves (*Canis lupus*), one black bear (*Ursus americanus*), and two grizzly bears (*Ursus arctos horribilis*) with a strain of *Br. suis* biovar 4 isolated from a sled dog from Alaska. Their experiments demonstrated that canids and ursids are susceptible to the infection via intraperitoneal inoculation and through oral mucous membranes. During acute stages of the infection, *Brucella* congregated in these species in high numbers in lymph nodes and distributed throughout the body. Importantly, *Brucella* invaded salivary glands and probably also mammary glands and kidney, thus providing conditions for shedding the bacteria in saliva, milk, and urine. The authors reported reproductive failure during infection in wolves, but were not confident that the failure was a consequence of the infection (47). Morton (48) experimentally infected foxes with *Br. suis* biovar 4 and observed that the incidence of positive titers, positive cultures, and shedding of bacteria was related to the number of *Brucella* organisms experimentally fed to the animals. Lowest doses did not produce infection. Highest doses produced positive titers and cultures.

Tests on rats showed transmission of *Br. abortus* biovar 1 from infected male to uninfected female rats resulted from sexual intercourse (49). Vertical transmission of *Br. abortus* caused sterility in pregnant mice (50); Wang et al. (51) documented vertical transmission of *Br. melitensis* on a pregnant mouse model. Guzman-Verri et al. (52) cited the more likely modes of transmission of *Br. ceti* to be through sexual intercourse, maternal feeding, aborted fetuses, placental tissues, vertical transmission from mother to the fetus or through fish or helminth reservoirs.

Brucellae have high viability and can survive in the environment for 3–21 days in spring-summer and for 151–233 days in winter-fall seasons. Brucellae maintain viability in carcasses (muscles, internal organs, and lymph nodes) at −7.2° to 38.4°C for 1–12 months (53). Long-term survival of *Br. microti* in soil was described and, thus, soil might act as a reservoir of infection (54).

## Prevalence of *Bartonella* Infections in Wild Carnivores

### General Prevalence Pattern

Overall, prevalence of *Bartonella* infections in carnivores was higher compared to *Brucella* infections. In the studies of over two Feliformia animals, the highest prevalence was registered by culture in bobcats [37%, 7/19, (55)], by IFA in bobcats again [74%, (56)], and by PCR of blood in Iberian lynx [33.3%, 10/30, (57)]. In studies of over two Caniformia animals, the highest *Bartonella* prevalence was registered by culture in gray foxes [49%, 26/53, (58)], by IFA in coyotes [89%, 48/53, (58)], and by PCR of blood in raccoons [43%, 16/37, (59)]. A very high overall prevalence of antibodies to *B. henselae* (95%) was detected among Brazilian free-ranging felids (60) (Table 1). As expected, the number of seropositive animals was usually higher than the numbers of culture or PCR positive individuals from the same study. Thus, of the 54 lions from South Africa, 5.2% were positive by culture, 3.7% were PCR positive for *Bartonella* DNA, and 17% had *Bartonella* antibodies (62). A study on golden jackals in Iraq found 14.5% of animals positive by PCR and 40.4% (23/57) by IFA (86).

**Table 1:** *Bartonella* studies in wild carnivores by species.

**Species**	**Location**	**Method**	**Prevalence*/Bartonella* spp**.	**References**
**SUBORDER FELIFORMIA**				
**Felidae family**				
Cheetah (*Acinonyx jubatus*)	Namibia	Culture, PCR (*16S rRNA, gltA, ribC, groEL, ftsZ*, ITS)	*Culture: 5.9% (1/17) new Bartonella strain between Bh and Bk*	(61)
	Africa	Culture, PCR (*gltA*), IFA	*Culture: 5.9% (1/17) unid'd Bartonella* sp. *close to Bk;* *PCR (blood): 23.3% (17/73); IFA 31.1% (23/74) B. henselae*	(62)
	Zimbabwe	Culture, PCR	*Culture: 33.3% (1/3) B. henselae*	(63)
Wildcat (*Felis silvestris*)	Spain	PCR (*gltA*, ITS)	*PCR (tissue): 16.7%(1/6) B. henselae*	(64)
		PCR (ITS)	*PCR (fleas): 16.7% (1/6 pools) B. alsatica*	(65)
Ocelot	Brazil	PCR	*PCR (blood): 0/7*	(66)
(*Leopardus pardalis*)		IFA	*IFA: 100% (1/1) B. henselae*	(60)
Little spotted cat (*Leopardus tigrinus*)	Brazil	IFA	*IFA: 100% (2/2) B. henselae*	(60)
Iberian lynx (*Lynx pardinus*)	Spain	PCR (ITS)	*PCR (fleas): 0% (0/5 pools)*	(65)
		PCR (*gltA*)	*PCR (blood): 13.3% (6/45) & 33.3% (10/30) B. henselae*	(57)
Bobcat (*Lynx rufus*)	Mexico	Culture, PCR (*gltA*, ITS)	*Culture: 0/5; PCR (blood): 0/5; PCR (fleas): 5.6% (1/18) Bartonella* sp.	(67)
	CA, USA	Culture, PCR (*16S rRNA, gltA, ribC, rpoB, ftsZ, groEL*, ITS), IFA	*Culture: 37% (7/19); Bh II and Bk* subsp. *bothieri; IFA:* *13/19 Bh II*	(55)
	CO, CA	ELISA	*ELISA: 31% Bartonella* sp.	(68)
	CA, USA	IFA	*IFA 74% (n = 25) B. henselae*	(56)
	USA	IFA	*IFA: 22.4% (19/85) B. henselae*	(17)
	Mexico		*IFA: 33.3% (2/6) B. henselae*	
	CA, USA	IFA	*IFA: 53% (33/62) B. henselae*	(69)
African lion (*Panthera leo*)	South Africa	Culture, PCR (*16S rRNA, gltA, ribC, groEL, ftsZ*, ITS)	*Culture: 5.2% (3/58) (2 Bh & 1 Bk subsp. koehlerae)*	(61)
	Zambia	PCR (ITS)	*PCR: 0% (0/24)*	(70)
	Africa	Culture, PCR (*gltA*), IFA	*Culture: 5.2% (3/58) 2 Bh & 1 unid'd Bartonella sp. close to Bk; PCR (blood): 3.7% (2/54); IFA: 16.8% (19/113) B. henselae*	(62)
	Africa	Culture, ELISA	*Culture: 1/65 (1.5%); B. henselae II; ELISA: 29% (18/62)*	(71)
	Zimbabwe	Culture	*Culture: 0%*	(63)
Far Eastern leopard	Russia	Western Blot	*WB: 0% (0/4) B. henselae*	(72)
(*Panthera pardus orientalis*)		Western Blot	*WB: 40% (2/5) B. henselae*	(73)
Amur tiger	Russia	Western Blot	*WB: 0% (0/17) B. henselae*	(72)
(*Panthera tigris altaica*)		Western Blot	*WB: 0% (0/17) B. henselae*	(73)
Iriomote cat (*Prionailurus*	Japan	PCR (ITS)	*PCR (ticks): 0% (0/13 pools), PCR (blood): 0% (0/11)*	(74)
*bengalensis iriomotensis*)		PCR (ITS)	*PCR (blood): 6% (2/33) B. henselae*	(75)
Tsushima leopard cat	Japan	PCR (ITS)	*PCR (ticks): 37.5% (3/8 cats), Bh; PCR (blood): 0% (0/6)*.	(74)
(*Prionailurus bengalensis*)		PCR (ITS)	*PCR (blood): 8% (1/13) Bc*	(75)
Mountain lion (*Puma concolor*)	FL, USA	PCR (ITS, *pap31, rpoB*)	*PCR: 100% (3/3) B. henselae*	(76)
	CA, USA	Culture, IFA, PCR (*16S rRNA, gltA, ribC, rpoB, ftsZ, groEL*, ITS)	*Culture: 29% (4/14) Bh II & Bk* subsp. *boulouisii; IFA: 8/14 Bh II*	(55)
	CO, USA	PCR (ITS, *gltA, ftsZ*)	*PCR (tissue): 0% (0/3)*	(77)
	CA, USA	IFA	*IFA: 37.1% (164/442) B. henselae I*	(78)
	CO, CA	ELISA	*ELISA: 16% Bartonella* sp.	(68)
	Brazil	IFA	*IFA: 88.9% (16/18) B. henselae*	(60)
	USA	IFA	*IFA: 20.2% (73/361) B. henselae total; 37.5% in coastal CA*	(17)
	Canada		*IFA: 0% (0/23) B. henselae*	
	Mexico		*IFA: 8.3% (1/12) B. henselae*	
	Central America, Venezuela		*IFA: 33.3% (8/24) B. henselae*	
	S. America		*IFA: 22.4% (11/49) B. henselae*	
	Andean countries		*IFA: 0% (0/10) B. henselae*	
	FL, USA	IFA	*IFA: 20% (7/35) B. henselae*	(79)
	CA, USA	IFA	*IFA: 35% (26/74) B. henselae*	(69)
**Herpestidae family**				
Egyptian mongoose (*Herpestes ichneumon*)	Algeria	PCR (ITS)	*PCR (tissue): 0% (0/1)*	(80)
Small Asian mongoose (*Herpestes javanicus*)	Grenada	IFA, PCR (*gltA, rpoB, 16S rRNA*)	*IFA: 32.3% (54/167); PCR (blood): 35.3% (18/51)* *B. henselae I*	(81)
	Japan	Culture, PCR (*16S rRNA, ftsZ, gltA, groEL, ribC,rpoB*)	*Culture: 15.9% (10/63) B. henselae*	(82)
**Hyaenidae family**				
Spotted hyena (*Crocuta crocuta*)	Zambia	PCR (ITS)	*PCR (blood): 0% (0/19)*	(70)
**Viverridae family**				
Common genet	Spain	PCR (ITS)	*PCR (blood): 5.9% (2/34) 2 Bc; PCR (ticks): 0% (0/15 pools)*	(83)
*(Genetta genetta)*		PCR (*gltA*, ITS)	*PCR (tissue): 0% (0/13)*	(64)
		PCR (ITS)	*PCR (fleas): 0% (0/10 pools)*	(65)
Masked palm civet (*Paguma larvata*)	Japan	Culture, PCR (*16S rRNA, ftsZ, gltA, groEL, ribC,rpoB*)	*Culture: 2.0% (1/50) B. henselae*	(82)
**SUBORDER CANIFORMIA**				
**Canidae family**				
Golden jackal (*Canis aureus*)	Serbia	PCR (ITS)	*PCR: 0% (0/216)*	(84)
	Israel	PCR (ITS, *ssrA, rpoB, gltA*)	*PCR: 13% (9/70) 5/9 Br, 3/9 related to Candidatus B. merieuxii, 1/9 between Bvb & B. merieuxii*	(85)
	Algeria	PCR (ITS)	*PCR (tissue): 0% (0/2)*	(80)
	Iraq	*PCR (ITS, rpoB, gltA), IFA*	*PCR: 12.3% (7/57) Candidatus B. merieuxii, 2% Bvb;* *IFA: 40.4% (23/57) any Bartonella spp., Bh 35% (20/57), Bc 37% (21/57), Bvb 33% (19/57), B. bovis 35% (20/57)*.	(86)
Coyote (*Canis latrans*)	Mexico	Culture, PCR (*gltA*, ITS)	*Culture: 1/18; PCR (blood): 5.6% (1/18) Br; 5.6% (1/18) Bvb; PCR (fleas): 15.1% (8/53) Bvb*	(67)
	CO, USA	PCR (ITS, *gltA, ftsZ*)	*PCR (tissue): 28% (7/25); 5/7 Bvb, 2/7 Br*	(77)
	CA, USA	PCR (*gltA*)	*PCR (valves, spleen): 21% (15/70); Bvb, Bh, Br*	(87)
	CA, USA	Culture, PCR (ITS, *gltA, rpoB, ftsZ, groEL*)	*Culture: 9.5% (2/21) Br*	(88)
	CA, USA	Culture, IFA, PCR (ITS, *gltA*)	*Culture: 42% (22/53) novel B. clarridgeiae-like; 9.4% (5/53) Bvb; IFA: 89% (48/53)*	(58)
	CA, USA	ELISA	*ELISA: 28% (n = 239) Bvb*	(89)
	CA, USA	IFA, PCR (*gltA, 16S rRNA*)	*IFA 76% (83/109) Bvb; PCR: 28% (31/109) Bvb*	(90)
	CA, USA	ELISA	*ELISA: 35% (306/869) Bvb (7–51% in CA)*	(91)
Wolf (*Canis lupus*)	Spain	PCR (*gltA*, ITS)	*PCR (tissue): 33.3% (1/3) Br*	(64)
Crab-eating fox	Brazil	PCR	*PCR (blood): 0/78*	(66)
(*Cerdocyon thous*)		PCR (*gltA, ribC*)	*PCR (fleas): 100% (9/9 fleas from the only fox), Br*	(92)
Darwin's fox (*Lycalopex fulvipes*)	Chile	PCR (ITS)	*PCR (blood): 0% (0/24)*	(93)
Wild dog (*Lycaon pictus*)	Zambia	PCR (ITS)	*PCR (blood): 0% (0/11)*	(70)
Raccoon dog	Korea	PCR (ITS, *groEL, rpoB*)	*PCR: 1.3% (2/152 spleen samples) B. henselae*	(94)
(*Nyctereutes procyonoides*)	Japan	PCR (ITS, *16SrRNA, ftsZ, gltA, groEL, ribC, rpoB*)	*PCR (blood): 0% (0/171)*	(95)
Gray fox	Mexico	Culture, PCR (*gltA*, ITS)	*Culture: 0/7; PCR (fleas): 9.7% (3/31) Br, 3.2% (1/31) Bvb*	(67)
*(Urocyon cinereoargenteus)*	CO, USA	PCR (ITS, *gltA, ftsZ*)	*PCR: 0/1*	(77)
	TX, USA	IFA	*IFA: 50% (66/132), 22Bc, 8 Bvb, 36 Bc+Bvb*	(96)
	CA, USA	PCR (ITS, *ftsZ*)	*PCR (fleas): 39% (42/108) (78.5% Br, 19% Bvb)*	(97)
		Culture, PCR (ITS, *gltA*), IFA	*Culture: 49% (26/53) (22/53 B. clarridgeiae-like, 5/53 Bvb); IFA: 89% (48/53) Bartonella spp*.	(58)
Island fox *(Urocyon littoralis)*	CA, USA	Culture, IFA, PCR (ITS, *pap31*)	*IFA: 62.7% (31.4% (16/51) Bc; 9.8% (5/51) Bvb; 21.6% (11/51) both); Culture: 11.8% (6/51) Bvb; PCR: 1 Bvb type III, 3 Br*	(98)
		IFA	*IFA: 25.8% (68/263) Bvb, 27.7% (73/263) Bc*	(99)
Arctic fox (*Vulpes lagopus*)	Canada	PCR	*PCR (blood): 15% (3/20) Bh*	(100)
Kit fox (*Vulpes macrotis*)	Mexico	Culture, PCR (*gltA*, ITS)	*Culture: 0/15; PCR (blood): 13.3% (2/15) Br; PCR (fleas): 5.0% (4/80) Br*	(67)
Red fox (*Vulpes vulpes*)	Slovakia	PCR	*PCR: 4.7% (19/407) fleas, Bartonella* spp.	(101)
	Romania	PCR (*ssrA*)	*PCR: 0/56*	(102)
	Austria	PCR (ITS)	*PCR (blood): 0% (0/351); PCR (spleen): 0.2% (1/506) Br*	(103)
	CO, USA	PCR (ITS, *gltA, ftsZ*)	*PCR: 27% (7/26) 2/7 Bvb, 5/7 Br*	(77)
	Israel	PCR (ITS, *ssrA, rpoB, gltA*)	*PCR: 18% (2/11) 1/2 Br, 1/2 related to B. merieuxii*	(85)
	Bosnia and Herzegovina	PCR (ITS)	*PCR: 0% (0/119)*	(104)
	Spain	PCR (ITS)	*PCR (blood): 25% (3/12) 3 Br;* *PCR (ticks): 0% (0/52 pools)*	(83)
		PCR (*gltA*, ITS)	*PCR: 1.6% (1/62) Br*	(64)
	Iraq	*PCR (ITS, rpoB, gltA), IFA*	*PCR: 0% (0/39); IFA: 13% (5/39) any Bartonella spp., Bh 5% (2/39), Bc 3% (1/39), Bvb 5% (2/39), B. bovis 13% (5/39)*.	(86)
	Australia	PCR (ITS, *gltA, 16S rRNA, ftsZ, rpoB*)	*PCR (fleas): 70.5% (24/34) (20/24 Bc, 4/24 Bh);* *PCR (blood): 1/14 Bc*	(105)
	France	Culture, PCR (ITS, *gltA, rpoB, ftsZ, groEL*)	*PCR: 100% (1/1) Br*	(88)
	Spain	PCR (ITS)	*PCR (fleas): 31.8% (7/22 pools), related to Br*	(65)
	Hungary	PCR (*groEL, pap31*)	*PCR (ticks): 0%; PCR (fleas): 4.2% (4/95 pools) Bartonella* spp.	(106)
**Mephitidae family**				
Hooded skunk (*Mephitis macroura*)	Mexico	Culture, PCR (ITS, *gltA*)	*Culture: 0/3; PCR (blood): 33.3% (1/3) Br;* *PCR (fleas): 26.7% (4/15) Br*	(67)
Striped skunk *(Mephitis mephitis)*	Mexico	Culture, PCR (ITS, *gltA*)	*Culture: 25% (2/8); PCR (blood): 12.5% (1/8) Br; 12.5% (1/8) Bvb; PCR (fleas): 5.4% (2/37) Br; 2.7% (1/37) Bvb*	(67)
	CO, USA	PCR (ITS, *gltA, ftsZ*)	*PCR: 23% (10/44) Br*	(77)
**Mustelidae family**				
Northern sea otter (*Enhydra lutris keyoni*)	AK, USA	IFA	*IFA: 34% (15/44) of live animals (27% Bw, 2.2% Bc, 4.5% Bc & Bw) and 50% of necropsied animals (14% Bw, 25% Bh & Bw, 2% Bh & Bc, 2% Bc & Bw, 6.2% Bh, Bc, & Bw)*	(107)
	AK, USA	Culture, PCR (ITS, *pap31, rpoB*)	*Culture: 0/9; PCR (valves): 45% (23/51); Bh I, B. bacilliformis, Bartonella* spp.	(108)
Southern sea otter (*Enhydra lutris nereis*)	CA, USA	IFA	*IFA: 16% (24/148) of necropsied animals (4.7% Bw, 1.3% Bc, 2% Bh, 5.4% Bh & Bw, 1.3% Bc & Bw, 1.3% Bh, Bc, & Bw)*	(107)
	CA, USA	Culture, PCR (ITS, *pap31, rpoB*)	*PCR (valves): 10% (3/30) B. spp, B. bacilliformis*	(108)
River otter (*Lontra canadensis*)	NC, USA	Culture, PCR (ITS)	*PCR: 15.2% (19/65), novel B. volans-like; culture: 1*	(109)
Beech marten	Spain	PCR (ITS)	*PCR (blood): 10% (1/10) 1 Bc; PCR (ticks): 0% (0/146 pools)*	(83)
(*Martes foina*)		PCR (*gltA*, ITS)	*PCR: 0% (0/26)*	(64)
Pine marten (*Martes martes*)	Spain	PCR (*gltA*, ITS)	*PCR: 0% (0/14)*	(64)
Japanese marten (*Martes melampus*)	Japan	PCR (ITS, *16SrRNA, ftsZ, gltA, groEL, ribC, rpoB*)	*PCR (blood): 12.5% (1/8) close to B. washoensis*	(95)
Japanese badger (*Meles anakuma*)	Japan	PCR (ITS, *16SrRNA, ftsZ, gltA, groEL, ribC, rpoB*)	*PCR (blood): 6.7% (1/15) novel Bartonella species*	(95)
European badger	Spain	PCR (*gltA*, ITS)	*PCR: 12% (9/75), B. clarridgeiae-like* sp.	(64)
(*Meles meles*)		PCR (ITS) (ITS, *16SrRNA, ftsZ, gltA, groEL, ribC, rpoB*)	*PCR (blood): 0% (0/3); PCR (ticks): 0% (0/2 pools)*	(83)
		PCR (ITS)	*PCR (fleas): 0% (0/3 pools)*	(65)
Stoat (*Mustela erminea*)	New Zealand	Culture, PCR (*gltA*)	*Culture (blood): 0% (0/47); PCR (blood): 0% (0/94)*	(110)
Japanese weasel *(Mustela itatsi)*	Japan	PCR (ITS, *16SrRNA, ftsZ, gltA, groEL, ribC, rpoB*)	*PCR (blood): 0% (0/2)*	(95)
Least weasel	Spain	PCR (*gltA*, ITS)	*PCR: 0% (0/5)*	(64)
(*Mustela nivalis*)	New Zealand	PCR (*gltA*)	*PCR (blood): 0% (0/2)*	(110)
European polecat (*Mustela putorius*)	Spain	PCR (*gltA*, ITS)	*PCR: 0% (0/5)*	(64)
Ferret (*Mustela putorius furo*)	New Zealand	Culture, PCR (*gltA*)	*Culture (blood): 0% (0/1); PCR (blood): 0% (0/25)*	(110)
Siberian weasel (*Mustela sibirica*)	Japan	Blood, PCR (ITS, *16SrRNA, ftsZ, gltA, groEL, ribC, rpoB*)	*PCR: 0% (0/1)*	(95)
American mink (*Mustela vison*)	Spain	PCR (*gltA*, ITS)	*PCR: 0% (0/3)*	(64)
American badger (*Taxidea taxus*)	Mexico	Culture, PCR (ITS, *gltA*)	*Culture: 0/6; PCR (blood): 0/6;* *PCR (fleas): 5.9% (2/34) Br; 2.9% (1/34) Bvb*	(67)
	CA, USA	IFA	*IFA: 10% (1/10) Bh; 10% (1/10) Bvb; 10% (1/10) Bh + Bc*	(111)
**Phocidae family**				
Harbor seal (*Phoca vitulina*)	The Netherlands	PCR (ITS, *rpoB*)	*PCR (spleen): 2.1% (1/48); PCR (lice): 16.7% (1/6 pools); 100% Bh / 97% B. grahamii*	(112)
**Procyonidae family**				
Ring-tailed coati (*Nasua nasua*)	Brazil	PCR	*PCR: 0/31*	(66)
Raccoon (*Procyon lotor*)	Mexico	Culture, PCR (ITS, *gltA*)	*Culture: 0/4; PCR (blood): 0/4; PCR (fleas): 0/17*	(67)
	CO, USA	PCR (ITS, *gltA, ftsZ*)	*PCR: 8% (14/186) Bartonella* spp. (21% Bvb, 79% Br)	(77)
	GA, USA	PCR (ITS)	*PCR (blood): 43% (16/37) Bartonella spp.: Bh (12/37), Bk (1/37)*	(59)
	Japan	PCR (ITS, *16SrRNA, ftsZ, gltA, groEL, ribC, rpoB*)	*PCR (blood): 0% (0/977)*	(95)
	CA, USA	Culture, PCR (ITS, *gltA, rpoB, ftsZ, groEL*)	*Culture: 26% (11/42) Br*	(88)
**Ursidae family**				
Black bear (*Ursus americanus*)	CO, USA	PCR (ITS, *gltA, ftsZ*)	*PCR: 0% (0/7)*	(77)

*Bc, B. clarridgeiae; Bh, B. henselae; Bk, B. koehlerae; Br, B. rochalimae; Bvb, B. vinsonii subsp. berkhoffii; Bw, B washoensis*.

### Age and Gender Pattern

Prior studies usually show no statistical difference in prevalence by age or gender in felines (61, 69, 79). However, Chomel et al. (17) found antibody prevalence for *B. henselae* to increase with age in pumas in California. In contrast, Rotstein et al. (79) found antibody prevalence higher in Florida panthers under 2 years of age (40%) compared to panthers over 2 years of age (13%).

### Geographic Pattern

Prevalence of *B. henselae* antibodies in mountain lions and bobcats varied significantly between different states of the U.S. (17). Mountain lions from Arizona, California, and Texas were more likely to be seropositive for *B. henselae* (26.7–40.0%) than pumas from the Northwest and Mountain states (0–11.8%) (17). In California, the highest prevalence in bobcats was from the coastal range (37.5%), while the highest prevalence in pumas was from Southern California and Sierra Nevada (17). The reported pattern was similar to the geographic distribution of *Bartonella* infection in domestic cats. It has been demonstrated that in cat populations (stray or pets), prevalence of infection was demonstrated to vary considerably with an increasing gradient from cold climates (0% in Norway) to warm and humid climates (68% in the Philippines) (14). In the U.S., prevalence of *B. henselae* antibodies in pet cats varied significantly with the highest average prevalence in the southeastern United States, Hawaii, coastal California, the Pacific Northwest, and low prevalence in Alaska, the Rocky Mountain-Great Plains region, and the Midwest (113). Comparing wild felids at four sites in California and Colorado, Bevins et al. (68) noted that seroprevalence varied considerably, but in almost all cases, it was higher in warmer and more humid California than in Colorado. For mountain lions, suburban land use predicted increased exposure to *Bartonella* species in southern California (114).

### Seasonal Pattern

Studies have yielded conflicting evidence about the seasonality of *B. vinsonii* subsp. *berkhoffii* infection in coyotes. First, Chang et al. (91) reported that the prevalence of *Bartonella* antibodies was highest in summer (42%) and lowest in spring (29%), whereas a geographically more restricted study conducted in coastal central California, U.S., by the same authors found the highest seroprevalence in winter (100%) and the lowest in summer (62%) (90). Investigating antibody prevalence in 239 coyotes from northern California, Beldomenico et al. (89) identified some environmental factors associated with the seropositivity. In that study, prevalence of antibodies against *B. vinsinii* subsp. *berkhoffii* was 44% in the summer, 40% in the spring, 27% in the winter, and 19% in the fall. The authors noticed that *Bartonella* seropositivity was associated with higher precipitation and proximity to the coast. In addition, coyotes seropositive for *B. vinsonii* subsp. *berkhoffii* were more likely to be seropositive for tick-borne agents *Anaplasma phagocytophilum* and mosquito-vectored *Dirofilaria immitis* (89). Interestingly, California Zoo felids of the genus *Felis* were found almost three times more likely to be seropositive for *B. henselae* than animals belonging to the genera *Panthera* and *Acinonyx* (69).

## Prevalence of *Brucella* Infections in Wild Carnivores

### General Pattern

Eighty-nine percent of *Brucella* studies of wild carnivores were conducted by serological and bacteriological methods, but no reports were found on culturing *Brucella* from representatives of suborder *Feliformia*. Only a few wild felid species (lion, jaguar, and bobcat), mongooses, and spotted hyena were serologically positive. Apart from one bobcat that had antibodies against *Br. canis* (115), the rest of seropositive Feliformia animals had antibodies against *Br. abortus*. We have to be cautious with the claim about presence of specific antibodies in this paper, as well in many other reports, because *Br. abortus* suspensions can also detect *Br. melitensis*. The highest seroprevalence was registered in white-tailed mongoose [33.3%, 1/3, (116)]. In evident contrast to Feliformia animals, prevalence of *Brucella* in various Caniformia species varied greatly, with many reporting high prevalences of positive antibody titeres. Antibodies to *Brucella* species were recorded in 40% of coyotes (117), 42% of wolves (118), 43% of black-backed jackals (116), 50% of Arctic foxes and 40% of red foxes (48), 64% of grizzly bears (119), 28% of Asian sea otters (120), 23% of California sea lions (121), and 74% of Australian seals (122). *Brucella* was cultured from 30.8% of wolves (123) (Table 2).

**Table 2:** *Brucella* studies in wild Carnivores by species.

**Species**	**Location**	**Method**	**Prevalence*/Brucella* spp**.	**References**
**SUBORDER FELIFORMIA**				
**Felidae family**				
Wildcat *(Felis silvestris)*	Russia	Serology	*Serology: 0/6*	(124)
Lynx *(Lynx canadensis)*	Canada	Serology, culture	*Serology/culture: 0*	(125)
Bobcat *(Lynx rufus)*	AL, USA	Culture	*Culture: 0/3*	(126)
	CA, USA	RPA	*Serology: 6.6% (5/75) B. abortus*	(127)
	TX, USA	RSA, SMTA	*Serology: 0 B. canis*	(128)
	USA	Tube agglutination	*Serology: 14% (1/7) B. canis (1/3 in TX, 0/1 in FL, 0/3 in SC)*	(115)
	UT, USA	Tube agglutination	*Serology: 0/3 B. abortus*	(129)
African lion (*Panthera leo*)	Tanzania	RBPT, BAPA, Riv	*Serology: 50% (1/2) Brucella* sp.	(130)
		Tube agglutination	*Serology: 15.4% (2/13) B. abortus*	(116)
	SAR	Agglutination	*Serology: 0/4 Brucella* sp.	(131)
Jaguar *(Panthera onca)*	Brazil	RBPT	*Serology: 3.2% (1/31) B. abortus*	(132)
		RBPT, 2-ME	*Serology: 0/11 B. abortus*	(133)
Leopard *(Panthera pardus)*	Tanzania	Tube agglutination	*Serology: 0/1 B. abortus*	(116)
Florida panther (*Puma concolor coryi*)	FL, USA	Plate agglutination	*Serology: 0/24 B. abortus*	(134)
**Herpestidae family**				
White-tailed mongoose (*Ichneumia albicauda*)	Tanzania	Tube agglutination	*Serology: 33.3% (1/3) B. abortus*	(116)
Banded mongoose (*Mungos mungo*)	Tanzania	Tube agglutination	*Serology: 1/1 B. abortus*	(116)
**Hyaenidae family**				
Spotted hyena *(Crocuta crocuta)*	SAR	Agglutination	*Serology: 0/2 Brucella* sp.	(131)
	Tanzania	Tube agglutination	*Serology: 26.7% (4/15) B. abortus*	(116)
	Tanzania	Agglutination	*Serology: 50% (2/4) Brucella* sp.	(135)
**Viverridae family**				
Genet *(Genetta genetta)*	Rhodesia	Tube agglutination	*Serology: 0/2 B. abortus*	(136)
Cape genet *(Genetta tigrina)*	Tanzania	Tube agglutination	*Serology: 0/3 B. abortus*	(116)
**SUBORDER CANIFORMIA**				
**Family Canidae**				
Golden jackal (*Canis aureus*)	Serbia	qPCR (*bcsp31, alkB, BMEI1162*)	qPCR: 1.9% (4/216) *B. canis*	(137)
Coyote (*Canis latrans*)	NC, USA	Card, RIV, IFA, agglutination	*Serology: 0/28 B. abortus/suis; 0/30 B. canis*	(138)
	NE, USA	Rapid slide agglutination	*Serology: 0/67 B. canis*	(139)
	WY, USA	Standard plate test	*Serology: 0/70 B. abortus and B. canis*	(140)
	GA, USA	Tube test	*Serology: 0/17 B. canis*	(141)
	TX, USA	Card, RIV, SAT,CF, ELISA	*Serology: CARD: 40.4% (38/94); RIV: 21.3% (20/94); CF: 22.3% (21/94); SAT: 18.1% (17.94); ELISA: 30.9% (29/94) B. abortus*	(117)
	AL, USA	Culture	*Culture: 0/2*	(126)
	TX, USA	BBA, RIV, SAT, CFT, culture	Serology: 18% (9/51) by 2+ tests. Culture: 16.3% (7/43) *B. abortus biovar 1*.	(142)
	CA, USA	Plate agglutination	*Serology:* 6% (9/148); *B. abortus*	(127)
	TX, USA	RSA, SMTA *B. canis*	Serology: Card: 5.6% (11/198); RSA: 6.6% (13/198); SMTA: 8.1% (16/198) ≥1:50 *B. canis*	(128)
	USA	Tube agglutination	*Serology: 2% (2/103) B. canis (2/86 in TX, 0/1* *- NY, 0/16 - ND)*	(115)
	TX, USA	Plate agglutination	*Serology: 0/33 B. abortus*	(143)
	UT, USA	Tube agglutination test	*Serology: 0/6 B. abortus*	(129)
Wolf (*Canis lupus*)	AK, USA	BBA, STT& SPT, CAR	*Serology: 0–25% B. suis biovar 4*	(144)
	Russia	Culture	*Culture: 11.8% (30/254) B. suis biovar 4*	(53)
	AK, USA	BAPA	*Serology: 1% (1/76) B. suis biovar 4*	(145)
	Canada	Culture	*Culture: 31% (4/13) B. abortus 1* [From (123)]	(146)
	Canada	Culture	*Culture: B. abortus biovar 1 isolated from a wolf. New strain of biovar 1 isolated from another wolf*.	(125)
	Canada	CF, rapid slide agglutination	*Serology: 0/3 B. abortus*	(147)
	AK, USA	SAT, CFT	*Serology: CF: 39% (11/28), agglutination: 26% (7/27) B. suis biovar 4*	(148)
	NY, USA	Tube agglutination	*Serology: 0/4 B. canis*	(115)
	Russia	Culture	*Culture: 15 Brucella* sp. *isolates*	(11)
	AK, USA	SAT, CFT	*Serology: 42.9% (3/7) B. suis biovar 4*	(118)
	Russia	Culture	*Culture: 10.9% (12/110) B. suis biovar 4*	(13)
	Russia	Serology	Serology: 0/56	(124)
Black-backed jackal *(Canis mesomelas)*	Tanzania	Tube agglutination test	*Serology: 43% (3/7) B. abortus*	(116)
Crab-eating fox	Brazil	RBPT, FPA	*Serology: 13.2% (5/38) smooth Brucella*	(149)
(*Cerdocyon thous*)	Brazil	RBPT, CFT	*Serology: 0/7 B. abortus*	(150)
	Bolivia	Slide agglutination/ AGID	*Serology: 0/5 B. canis*	(151)
		Serology	*Serology: 0/55 B. canis*	(152)
Maned wolf *(Chrysocyon brachyurus)*	Brazil	RBPT, CFT	*Serology: 0/3 B. abortus*	(150)
Foxes?	Argentina	Characterization	*B. abortus*	(153)
Patagonian gray fox *(Dusicyon griseus griseus)*	Argentina	Plate agglutination test	*Serology: 21.7% of 318 (11.3% of these ≥1:100); B. abortus*	(154)
Pampas gray fox *(Dusicyon gymnocercus antiquus)*	Argentina	Plate agglutination test, culture	*Serology: 25.4% of 410 (13.9% of these ≥1:100); Culture: 16.1% (5/31 pools of 77 foxes), B. abortus biovar 1*.	(154)
Pampas fox (*Lycalopex gymnocercus*)	Bolivia	Slide agglutination/ AGID	*Serology: 0/9 B. canis*	(151)
Hoary fox (*Lycalopex vetulus*)	Brazil	BPAT, AGID, MAT, SMTA	*Serology: BPAT: 26.6% (16/60) B. abortus; SMTA: 6.7% (4/60); AGID: 0/60 B. canis*	(155)
Wild dog *(Lycaon pictus)*	Tanzania	Tube agglutination test	*Serology: 33.3% (1/3) at 1:160 B. abortus*	(116)
Bat-eared fox (*Otocyon megalotis*)	Tanzania	Tube agglutination test	*Serology: 0/1 B. abortus*	(116)
Gray fox (*Urocyon cinereoargenteus)*	AL, USA	CARD, STA, 2-ME, RIV	*Serology: 14.3% (1/7) during exposure, or 5.6% (1/18) total; B. abortus biovar 1*	(126)
	FL, SC, USA	Tube agglutination	*Serology: 0/15 (0/10 in FL, 0/5 in SC) B. canis*	(115)
	AR, USA	Culture	*Culture: 0/14*	(156)
Arctic fox (*Vulpes lagopus*)	Russia	Culture	*Culture: 2.3% (18/777) B. suis biovar 4*	(53)
	AK, USA	SP, BBA, Riv, ST, ME, CF, culture	*Serology: 50% (2/4), culture: 25% (1/4) B. suis biovar 4*	(48)
	Russia	Culture	*Culture: 10 Brucella isolates*	(11)
	Russia	Culture	*Culture: 1.1% (4/370) B. suis biovar 4*	(13)
	Russia	Culture	*Culture: 1.7% (9/530) B. suis biovar 4*	(157)
	Russia	Culture, serology	*Culture: 4% (5/128); Serology: 3% (58/1,890)*	(124)
Kit fox *(Vulpes macrotis)*	UT, USA	Tube agglutination test	*Serology: 0/5 B. abortus*	(129)
San Joaquin kit fox	CA, USA	Serology	*Serology: 0/46 B. canis*	(152)
*(Vulpes macrotis mutica)*	CA, USA	CF, BBA, SAT, 2-ME	*Serology: B. abortus CF: 8% (3/23) in 1981/2, card test 3% (1/29) in 1984; B. canis CF: 14% (5/23) in 1981/2, ME: 0% (0/20) in 1984*.	(158)
Red fox (*Vulpes vulpes*)	Austria	Characterization	*B. vulpis* sp. *nov*.	(159)
	Austria	Culture	*Two novel Brucella strains*	(160)
	Austria	Culture	*Culture: B. microti*	(161)
	AK, USA	SP, BBA, Riv, ST, ME, CF, culture	*Serology: 39.5% (15/38), culture: 8% (3/38) B. suis biovar 4*	(48)
	Canada	Culture	*Culture: 2.7% (1/37) B. abortus biovar 1* [from (123)]	(146)
	Canada	Culture	*Culture: B. abortus biovar 1*	(125)
	AK, USA	STT, CFT	*Serology: CF: 18.2% (2/11); agglutination: 9.1% (1/11); B. suis biovar 4*	(148)
	NY, USA	Tube agglutination	*Serology: 1.5% (1/68) B. canis*	(115)
	Wales, UK	RBPT, SAT, CFT, AGT	*Serology: 9% (8/87); culture: B. abortus biovar 1*	(162)
	Ireland, UK	SAT, CF, culture	*Serology: SAT: 12.5% (4/32) B. abortus; Culture: 0/2*	(163)
	AR, USA	Culture	*Culture: 0/9*	(156)
	Russia	Serology, culture	Serology: 8.5% (374/4,380); culture: 7.8% (13/166);	(124)
	Bulgaria	Serology, culture	Serology: 3.6% (16/440); culture: 3.5% (1/29) *B. suis*	(164)
**Family Mephitidae**				
Striped skunk *(Mephitis mephitis)*	CA, USA	Plate agglutination	*Serology: 8.7% (2/23) ≥ 1:100 B. abortus*	(127)
	TX, USA	RSA, SMTA	*Serology: 0 B. canis*	(128)
	USA	Tube agglutination ≥1:200	*Serology: 0/17 B. canis*	(115)
	AR, USA	Culture	*Culture: 0/18*	(156)
Western spotted skunk (*Spilogale gracilis*)	CA, USA	Plate agglutination	*Serology: 3.85% (1/26) ≥ 1:100 B. abortus*	(127)
**Family Mustelidae**				
Northern sea otter	WA, USA	BAPA, ELISA, FPA	*Serology: 10% (3/30) B. abortus*	(165)
(*Enhydra lutris keyoni*)	AK, USA	cELISA	*Serology: 2.7% (1/72) marine Brucella* sp.	(120)
	WA, USA	Card, BAPA, rivanol, CF	*Serology: 0/30 B. abortus*	(166)
	AK, USA	RBT	*Serology: 7.7% (5/65) B. abortus*	(167)
Asian sea otter (*Enhydra lutris lutris)*	Russia	PCR (*IS711*)	*PCR (rectal swabs): 4% (3/78) B. abortus, B. melitensis, B. pinnipedialis*	(168)
		ELISA	*Serology: 28.1% (25/89) marine Brucella* sp.	(120)
Southern sea otter *(Enhydra lutris nereis)*	CA, USA	Culture, ELISA, FPA, PCR (*16S rDNA, bp26*)	*1/1 marine Brucella* sp.	(169)
	CA, USA	RBT	*Serology: 5.9% (4/68) B. abortus*	(167)
Wolverine *(Gulo gulo)*	Russia	Culture	*Culture: 10.2% (4/39) B. suis biovar 4*	(53)
		Culture	*Culture: 1 B. suis biovar 4*	(11)
		Culture	*Culture: 11.1% (1/9) B. suis biovar 4*	(13)
Eurasian otter *(Lutra lutra)*	UK	ELISA, culture	*Serology: 10.8% (8/74) B. abortus; culture: 0.6% (1/160) marine Brucella* sp.	(170)
	UK	Culture	*Culture: 1/1 Brucella* sp.	(171)
American pine marten (*Martes americana*)	Canada	Serology, culture	*Serology/culture: 0*	(125)
Asian badger (*Meles leucurus)*	South Korea	PCR, culture	*PCR (tissue): 100% (1/1) Brucella* sp.; *culture: 0/1*	(172)
Stoat	Russia	Culture	*Culture: 1.2% (6/484) B. suis biovar 4*	(53)
(*Mustela erminea*)		Serology, culture	*Serology: 0/7; culture: 0/3*	(124)
Steppe polecat (*Mustela eversmanii*)	Russia	Serology, culture	*Serology: 0/30; culture: 0/15*	(124)
European mink (*Mustela lutreola*)	Russia	HT, AT, CFT	*Serology: 7.2% (108/1,506); culture: 10.4% (11/106)*	(124)
Least weasel (*Mustela nivalis*)	France	Culture	*Culture: 0/10*	(173)
American mink (*Neovison vison)*	Argentina	ELISA, CFT	*Serology: 9.2% (8/87) B. abortus*	(174)
Fisher (*Pekania pennanti*)	Canada	Serology and culture	*Serology/culture: 0*	(125)
American badger *(Taxidea taxus)*	CA, USA	Plate agglutination	*Serology: 50% (2/4) B. abortus*	(127)
	TX, USA	RSA, SMTA	*Serology: B. canis*	(128)
	UT, USA	Tube agglutination	*Serology: 0/5 B. abortus*	(129)
	UT, USA	CF; agglutination test	*Serology: 0/1 B. canis*	(175)
**Family Procyonidae**				
Brown-nosed coati (*Nasua nasua)*	Brazil	RBPT, FPA	*Serology: 8.8% (3/34) smooth Brucella*	(149)
Raccoon (*Procyon lotor*)	S Korea	ELISA, PCR, culture	*Serology: 0/32; PCR (blood): 11.1% (1/9); culture: 0/9;* *PCR (tissue): 40% (2/5); culture: 0/5. B. abortus*	(172)
	NE, USA	Rapid slide agglutination	*Serology: 0% (0/63) B. canis*	(139)
	AL, USA	Culture, CARD, STA, 2-ME, RIV	*Culture: 16.7% (1/6) B. abortus biovar 1 during exposure, or 4.2% (1/24) total; Serology: 25% (1/4) during exposure, 8.3% (1/12) post exposure, or 9.5% (2/21) total*	(126)
	TX, USA	SAT, card, CF	*Serology: 0/3 B. abortus*	(176)
	AL, USA	Culture, card, tube agglutination test	*Culture: B. abortus biovar 1 from spleen & lymph node; Serology: Card: trace; tube: 1:200*	(177)
	CA, USA	Plate agglutination	*Serology: 6.25% (1/16) ≥ 1:100 B. abortus*	(127)
	TX, USA	RSA ≥1:2, SMTA	Serology: Card: 9.1% (1/11); RSA: 27.3% (3/11); SMTA: 9.1% (1/11)≥1:50, 9.1% (1/11)≥1:100; 0% (0/11)≥1:200 *B. canis*	(128)
	FL, USA	Tube agglutination	*Serology: 0.3% (1/360 (0.4% (1/269) in FL, 0/87 in TX, 0/4 in SC)) B. canis*	(115)
	FL, USA	Agglutination test	*Serology: 1.8% (4/222) at two counties (0.7 and 3.9%), B. canis*	(115)
	AR, USA	Culture	*Culture: 0/25*	(156)
**Family Ursidae**				
Black bear (*Ursus americanus*)	MD, USA	BAPA/card	*Serology: 0% (0/61) B. canis*	(178)
	AK, USA	BBA, SPT	*Serology: 0.8% (1/92)*	(179)
	Canada	CF, rapid slide agglutination	*Serology: 0.4% (1/283) B. abortus*	(147)
	ID, USA	Tube agglutination	*Serology: 5% (18/332) ≥1:20 B. abortus*	(180)
Brown bear *(Ursus arctos)*	AK, USA	BBA, SPT	*Serology: 14% (13/92)*	(179)
Alaska peninsula brown bear *(Ursus arctos gyas)*	AK, USA	ELISA, RBPT, ELISA+, RBPT+	*Serology: ELISA: 0/6; RBPT: 83.3% (5/6);* *ELISA+: 0/6; RBPT+: 33.3% (1/6); B. abortus*	(119)
Grizzly bear *(Ursus arctos horriblis)*	AK, USA	ELISA, RBPT, ELISA+, RBPT+, *B. abortus*	*Serology: ELISA: 62.1% (36/58); RBPT: 70.7% (41/58); ELISA+: 63.8% (37/58); RBPT+: 69% (40/58); B. abortus*	(119)
		BBA, STT& SPT ≥1:50, CAR	*Serology: 0–24% B. abortus*	(144)
		SP, BBA, Riv, ST, ME, CF	*Serology: 25% (2/8)*	(48)
		SPT, card	*Serology: 5% (6/122)*	(181)
		SAT, CFT	*CF: 29% (6/21); SAT: 43% (9/21) at Porcupine caribou herd; CF: 94% (15/16); SAT: 82% (14/17) at Arctic caribou herd*.	(148)
Kodiak brown bear *(Ursus arctos middendorffi)*	AK, USA	ELISA, RBPT, ELISA+, RBPT+	*Serology: ELISA: 75% (6/8); RBPT: 87.5% (7/8);* *ELISA+: 75% (6/8); RBPT+: 75% (6/8) B. abortus*	(119)
Marsican brown bear (*Ursus arctos marsicanus)*	Italy	Rapid serum agglutination	*Serology: 10% (2/22) B. abortus / B. melitensis*	(182)
Polar bear	AK, USA	BBA, SPT	*Serology: 13% (18/138)*	(183)
*(Ursus maritimus)*		BBA, SPT, cELISA	*Serology: 10.2% (28/275) (6.8% –18.5% over 2003–2006)*	(179)
		BACA, rapid automated presumptive test	*Serology: 5% (25/500) B. abortus*	(184)
		SAW, SAW-EDTA, RBT, Protein-A ELISA	*Serology: 5.4% (16/297) by all tests;* *SAW: 6% (18/297); SAW-EDTA: 5.4% (16/297); RB: 7% (21/297); Protein-A ELISA 53% (157/297)*	(185)
**SUPERFAMILY PINNIPEDIA**				
**Family Odobenidae (Walruses)**				
Pacific walrus (*Odobenus rosmarus divergens*)	AK, USA	Card, tube agglutination	*Serology: 0/40 B. abortus*	(186)
Atlantic walrus	Canada	ELISA	*Serology: 2.9% (5/170) B. abortus*	(187)
*(Odobenus rosmarus rosmarus)*	Canada	ELISA, tube agglutination	*ELISA: 12% (7/59); tube test: 5/5 of ELISA positive*	(188)
**Family Otariidae (Fur seals & sea lions)**				
South American fur seal (*Arctocephalus australis*)	Peru	ELISA, PCR	*Card: 0/29 B. canis; 3.5% (1/29) B. abortus; ELISA: marine Brucella 53.7% (15/28)*	(189)
New Zealand fur seal *(Arctocephalus forsteri)*	New Zealand	ELISA	*Serology: 0/101 (pre-weaned pups) B. abortus*	(190)
Antarctic fur seal	Antarctica	RBT, ELISA	*Serology: 0% (0/21) B. abortus*	(191)
(*Arctocephalus gazella*)		RBT, ELISA	*Serology: 0/64 B. abortus*	(192)
		ELISA	*Serology: 7.7% (4/52)*	(193)
		RBT, ELISA, COMPELISA	*Serology: RBT: 1.2% (1/86); ELISA: 5.8% (5/86)*	(194)
		RBT, CFT, AGID, ELISA	*Serology: 0–31% (AGID 0/16; RBT 1/16; CFT 2/16; ELISA 5/16) B. abortus*	(195)
Australian fur seal (*Arctocephalus pusillus doriferus*)	Australia	ELISA, FPA	*ELISA: 57% (71/125) adult females; 74% (32/43) in 2007; 53% (32/61) in 2008; 33% (7/21) in 2009*.	(122, 196)
		ELISA	*Serology: 7% (1/15)*	(197)
Guadalupe fur seal (*Arctocephalus townsendi)*	Mexico	RBT, RIV, FPA	*Serology: 0/46 (pups 1-2 mo old) B. abortus*	(198)
Northern fur seal	AK, USA	ELISA	*Serology: 0/107*	(199)
*(Callorhinus ursinus)*		qPCR (*IS711*), BMAT	*qPCR (placentas): 5% (6/119); PCR (sera): 1/40;* *BMAT: 1/40 positive, 12/40 borderline*	(200)
Steller sea lion	AK, USA	ELISA	*Serology: 1.6% (2/124)*	(199)
(*Eumetopias jubatus*)		ELISA	*Serology: 0.5% (1/197)*	(201)
Western Steller's sea lion (*Eumetopias jubatus jubatus*)	Japan	ELISA, *Western blot*	*Serology: 18% (3/17) B. abortus; 18% (3/17) B. canis*	(202)
Australian sea lion (*Neophoca cinerea*)		ELISA	*Serology: 75% (9/12)*	(197)
New Zealand sea lion (*Phocarctos hookeri*)	New Zealand	ELISA	*Serology: 0.7% (1/147) B. abortus*	(203)
California sea lion (*Zalophus californianus)*	CA, USA	RBT, AGID *B. abortus*, FPA, PCR (*bp26*), culture	*Serology: 22.7% (5/22) Brucella spp.; culture: 0%;* *2/5 strains of terrestrial origin*	(121)
		Culture, PCR (*omp2, bcsp31*)	*PCR: 5.1% (3/59) placentae, culture: 3.4% (2/59)*	(204)
**Family Phocidae (True seals)**				
Hooded seal *(Cystophora cristata)*	Norway	RBT, ELISA	*Serology: 0/3*	(205)
	Norway	ELISA	*Culture: 5% (1/21) B. pinnipedialis from lymph node; Serology: overall 15.6% (59/379) (pups 2.5%, yearlings 35.3%)*	(206)
	Norway	SAW-EDTA, RB, CFT, ELISA, PCR, culture	*Serology: 31% (9/29), culture: 38% (11/29) B. pinnipedialis; highest in spleen (9/29) and lung lymph nodes (9/24)*	(207)
	UK	Culture	*Culture: from 3 seals from lung, spleen, lymph nodes etc*.	(208)
	Canada	ELISA	*Serology: 4.9% (10/204) B. abortus*	(187)
	UK	ELISA, culture	*Serology: 50% (1/2); culture: 60% (3/5) B. abortus*	(170)
		*ELISA, SAT, SAT-EDTA, RBT, CFT*	*Serology: 35% (48/137) B. abortus*	(209)
	UK	Culture	*Culture: 1/1*	(171)
Bearded seal		Culture, ELISA	*Culture: B. pinnipedialis; serology: 11% (22/200)*	(210)
(*Erignathus barbatus)*	AK, USA	Card, tube agglutination	*Serology: 0/6 B. abortus*	(211)
		*ELISA, SAT, SAT-EDTA, RBT*	*Serology: 0/16 B. abortus*	(209)
Ribbon seal	AK, USA	*ELISA*	*Serology: 16.4% (9/55)*	(199)
(*Histriophoca fasciata)*	Japan	ELISA *B. abortus, B. canis, Western blot*	*Serology: 15% (3/20) B. abortus; 5% (1/20) B. canis*	(202)
Gray seal (*Halichoerus grypus*)	The Netherlands	RBT, SAT, ELISA	*Serology: SAT 9% (1/11), ELISA 36% (4/11) B. abortus*	(212)
	Finland	Culture, PCR	*Culture: 2.5% (3/122 livers) B. pinnipedialis; PCR: Brucella DNA in liver flukes 1/4 seals*	(213)
	Germany	Culture, PCR	*Culture: 3% (1/34 lungs)*	(214)
	UK	Culture	*Culture: 3/3 from lungs, testes, spleen*	(208)
	Canada	ELISA	*Serology: 3.9% (10/255) B. abortus*	(187)
	UK	ELISA, culture	*Serology: 19% (24/125) B. abortus; culture: 3% (2/66)*	(170)
	UK	ELISA	*Serology: 10% (6/62)* marine *Brucella*	(215)
	UK	Culture	*Culture: 6.3% (1/16 testes)*	(171)
	UK	RBPT, SAT, ELISA	*Serology: RBPT: 32% (10/31), SAT: 13% (4/31); ELISA: 23% (7/31) B. abortus*	(216)
Leopard seal (*Hydrurga leptonyx*)	Australia	ELISA	*Serology: 33% (1/3)*	(197)
Weddell seal	Antarctica	ELISA, RBT	*Serology: ELISA: 24.2% (8/33); RBT 65.6% (21/33) B. abortus*	(191)
(*Leptonychotes weddellii)*		RBT, ELISA	*Serology: 37% (7/19) B. abortus*	(192)
		RBPT, CFT, SAT, ELISA, culture	*Serology: RBPT: 62.9% (22/35); SAT: 68.6% (24/35); CFT: 98.3% (56/57); ELISA: 96.5% (55/57); culture: 0*	(217)
		Unspecified	*Serology: 0/81*	(218)
		RBT, ELISA, COMPELISA	*Serology: 42% (5/12)*	(219)
		RBT, CFT, AGID, ELISA	*Serology: 0–100% (RBT 0/1; AGID 0/1; CFT 0/1; ELISA 1/1) B. abortus*	(195)
Crab-eater seal (*Lobodon carcinophaga*)		RBT, ELISA	*Serology: 11% (1/9) B. abortus*	(192)
Southern elephant seal	Antarctica	ELISA, RBT	*Serology: 4.7% (2/48) B. abortus*	(191)
(*Mirounga leonina*)		ELISA	*Serology: 0/13*	(193)
Hawaiian monk seal (*Neomonachus schauinslandi)*	HI, USA	qPCR (*IS711*)	*PCR (placenta): 0/50*	(220)
	HI, USA	SCA, PCFIA, BAPA, CF, SPT, RIV	*Serology: B. canis: 0/111; B. abortus: SCA: 5–33%*	(221)
		BPAT, ELISA, FPA	*Serology: all tests: 17.1% (28/164); BPAT: 17.1% (28/144), cELISA: 15.2% (25/144), iELISA and FPA: 11.6% (19/144)*.	(222)
Ross seal (*Ommatophoca rossii*)		RBT, ELISA	*Serology: 5% (1/20) B. abortus*	(192)
Harp seal	Norway	RBT, C-ELISA	*Serology: 0/6*	(205)
(*Pagophilus groenlandicus*)	NE, USA	Culture, card, BAPA, RIV	*Serology: 8% (4/53); culture: 33.3% (3/9) from lungs and lymph nodes, B. abortus*	(223)
	Canada	ELISA	*2% (8/453) (1.8% (8/453), 1.8% (5/269), 3.1% (3/95)) B. abortus*	(187)
	UK	ELISA	*Serology: 50% (1/2) B. abortus*	(170)
	Canada	Culture	*Culture: 1/1 from lymph nodes - novel Brucella* sp.	(224)
		*ELISA, SAT, SAT-EDTA, RBT, CFT*	*Serology: 2% (15/811)*	(209)
Ringed seal *(Phoca hispida)*	Sweden	RBT, C-ELISA	*Serology: 16.7% (2/12)*	(205)
	AK, USA	ELISA	*Serology: 14% (21/150)*	(199)
	Norway	SAW-EDTA, RB, CFT, ELISA	*Serology: 0/20 B. abortus*	(207)
	Canada	ELISA	*Serology: 1.1% (7/628) B. abortus*	(187)
	UK	ELISA	*Serology: 0/1 B. abortus*	(170)
	Canada	Culture	*Culture: 66.7% (4/6) from lymph nodes - novel Bartonella* sp.	(224)
		*ELISA, SAT, SAT-EDTA, RBT, CFT*	*Serology: 10% (5/49) B. abortus*	(209)
	Canada	ELISA	*Serology: 4% (10/248)*	(188)
Spotted seal (*Phoca largha)*	AK, USA	ELISA	*Serology: 18.8% (16/85)*	(199)
	Japan	ELISA, *Western blot*	*Serology: 66% (27/41) B. abortus; 32% (13/41) B. canis*	(202)
Baikal seal *(Phoca sibirica)*	Russia	RBPT, SAT, iELISA	*Serology: 0/45*	(216)
Harbor seal *(Phoca vitulina)*	The Netherlands	Culture, RBT, SAT, ELISA, qPCR (*IS711*)	*Serology: RBT 53% (21/40), SAT 40% (16/40), ELISA 60% (24/40); qPCR (tissue) 77% (30/39); culture: 31% (12/39)*	(212)
	AK, USA	ELISA	*Serology: 24.6% (276/1,122)*	(199)
	Germany	Culture	*Culture: 17% (0–25% in different years)*	(225)
	UK	RBT, ELISA, culture	*Serology: RBT: 15.9% B. abortus, cELISA: 25.4% (n = 343) B.melitensis; culture: 16% (24/150)*	(226)
	AK, USA	cELISA	*Serology: 52% of 152 adults, 53% of 110 subadults, 77% of 93 yearlings, 26% pups <5 mo old (n = 554), from 29% to 64% in different populations; marine Brucella* sp.	(227)
	AK, USA	Card, plate, ELISA, RSAT	*Serology: 16–74% (plate B. abortus: 74%; card B. abortus: 16%; cELISA marine Brucella: 37%; ELISA B. ovis and RSAT: 0)*	(228)
	Germany	Culture	*Culture: 11% (47/426) from lungs and lung lymph nodes*	(214)
	WA, USA	Serology, PCR, culture	*Serology: 7% pups, 8% adults, 34% subadults, 54% weaned pups/yearlings*	(229)
	USA	Card, BAPA, RIV, culture	*Serology: 14% (3/21) B. abortus; culture: 2/4 from lungs and lymph nodes*	(223)
	UK	Culture	*Culture: from 11 animals from lung, spleen, lymph nodes*	(208)
	Canada, USA	ELISA	*Serology: 12.9% (21/163) (US Atlantic coast 50% (4/8)) B. abortus*	(187)
	UK	ELISA, culture	*Serology: 49% (147/297) B. abortus; culture: 10/117*	(170)
	UK	ELISA	*Serology: 8% (1/12) B. melitensis*	(215)
	UK	Culture	*Culture: 14.3% (4/28)*	(171)
	UK	Culture, RBPT, SAT, ELISA	*Culture: 4 (2 spleens, 2 lymph nodes) Brucella* spp.; *Serology: RBPT: 49% (69/140), SAT: 18% (25/140), iELISA: 32% (45/140)*	(216, 230)
Western Pacific harbor seal *(Phoca vitulina stejnegeri)*	Japan	ELISA	*Serology: 24% (13/55) B. abortus; 11% (6/55) B. canis*	(202)
Pacific harbor seal *(Phoca vitulina richardsi)*	WA, USA	BAPA, BBA, qPCR (*bcsp31*), CF, RIV, culture	*Culture: 17.7% (18/102); qPCR: 1.2% (4/336); Serology: 7.6% (100/1314 live healthy seals)*	(231)
	AK, USA	ELISA	*Serology: 46% (46/100) Brucella* spp.	(232)
		BAPA, BBA, CF, culture	*BAPA, BBA, CF: 1/1; Culture: 1/1 from lung and lymph nodes. Brucella* spp. *in lungworms*.	(233)

*AGID, Agar gel immunodiffusion; BACA, buffered acidified card antigen; BAPA, buffered acidified plate antigen; BBA, buffered Brucella antigen test; BMAT, Brucella microagglutination test; BPAT, Buffered antigen plate agglutination test; CF, Complement fixation; CFT, the cold complement fixation tube test; RAS, Rapid slide agglutination; RBPT, Rose Bengal Plate test; RIV, the rivanol precipitation test; RPA, Rapid Plate Agglutination; RSA, Rapid Slide Agglutination; SAT, standard agglutination tube test; SAW, Slow Agglutination of Wright; SCA, Standard Card Agglutination test; SMTA, salt 2-mercaptoethanol tube agglutination test; SPT, Standard plate test; STT, Standard test tube*.

### Age Pattern

We could find information on age dependence only in marine *Brucella*. In the 2018 study on gray and harbor seals, Kroese et al. (212) noted remarkable age-dependent prevalence of *Br. pinnipedialis* in both serology and in the investigation of the tissues from stranded animals. The PCR positivity was 84% (26/31) in juveniles compared to 57% (4/7) in adults and *Br. pinnipedialis* was cultured only from juveniles and not from adults in that study. Similar age dependence was shown in harbor seals by Miller et al. (169) and Ewalt et al. (229). Nymo et al. (206) noted the age-dependent prevalence of anti-*Brucella* antibodies in hooded seals. Pups (<1 mo old) had a substantially lower probability of being seropositive (4/159, 2.5%) than yearlings (6/17, 35.3%), suggesting that exposure may occur post-weaning, during the first year of life. For seals over 1 year old, the mean probability of being seropositive decreased with age, with no seropositives older than 5 years, indicating loss of antibody titer with either chronicity or clearance of infection (206).

## *Bartonella* Species Identified in Wild Carnivores

### *Bartonella* Species in Wild Feliformia Animals

Wild Feliformia animals mostly carry the same *Bartonella* species as domestic cats, namely *B. henselae* (types I and II), *B. koehlerae*, and *B. clarridgeiae* (234). The same species were detected in feral cats from Georgia, U.S. (59). In Africa, free-ranging lions were found infected with *B. henselae* type II and *B. koehlerae* subsp. *koehlerae* and Namibian cheetah with a strain that clustered between *B. henselae* and *B. koehlerae* and was considered a new subspecies of *B. koehlerae* (61, 63). In Japan, *B. henselae* was found in Iriomote leopard cats and *B. clarridgeiae* DNA was detected in Tsushima leopard cats (74, 82).

In a study on free-ranging mountain lions and bobcats from California, U.S., Chomel et al. (55) described new *Bartonella* strains, which were similar to but different from *B. henselae* and *B. koehlerae*, and named them *B. koehlerae* subsp. *boulouisii* and *B. koehlerae* subsp. *bothieri*. Phylogenetic analysis based on comparison of four genetic markers revealed two clusters: one with five strains obtained from bobcats and another with three strains obtained from mountain lions indicating a degree of host-speciation of these strains (55). In Brazil, sequencing analysis revealed a *Bartonella* strain close to but different from *B. henselae* and *B. koehlerae* in wild-born captive margay (*Leopardus wiedii*) (235).

Other *Bartonella* species were detected in fleas collected from wild felids. For example, *Bartonella alsatica* was found in one of six rabbit fleas *Spilopsyllus cuniculi* collected from a European wildcat (*F. silvestris)* in Spain (65). This *Bartonella* species is usually associated with rabbits and possibly fleas were infected or they contained blood meal from infected rabbits, as *S. cuniculi* is normally found on European rabbits (*Oryctolagus cuniculus*). A different situation has been reported by López-Pérez et al. (67) regarding a genetic variant obtained from a flea (*Pulex simulans*) collected from a bobcat (*L. rufus)* in northwestern Mexico. This variant had ITS sequence 99.1% similar to a strain previously isolated from another bobcat from California, U.S., but distant from all other *Bartonella* genotypes.

### *Bartonella* Species in Wild Caniformia Animals

In the studies, Caniformia animals were found to carry *B. henselae, B. clarridgeiae, B. vinsonii* subsp. *berkhoffii, B.rochalimae, B. washoensis*, and *B. bacilliformis*. In an investigation of wild carnivores from Colorado, U.S., Bai et al. (77) identified two *Bartonella* species, *B. vinsonii* subsp. *berkhoffii* and *B. rochalimae*. Striped skunks exclusively carried *B. rochalimae*, while coyotes, red foxes, and raccoons were infected with either or both *Bartonella* species. *Bartonella rochalimae* DNA was found in a wolf *(C. lupus*) in northern Spain (64). Investigating wild canids along with stray dogs throughout Iraq, Chomel et al. (86) identified a novel strain of *Bartonella*, which was named *Candidatus* B. merieuxii, in six jackals (*Canis aureus*). By three genetic markers, the “jackal” strain was aligned most closely with *B. bovis* and the other ruminant *Bartonella* species. Sequences closely related to *Candidatus* Bartonella merieuxii later were found in three jackals and one red fox (*V. vulpes*) in Israel (85). Besides this strain, *B. rochalimae* and *B. rochalimae*-like were found in five jackals and one fox, and one jackal harbored *B. vinsonii* subsp. *berkhoffii* (85).

Kehoe et al. (87) documented the presence of three *Bartonella* species in heart valves and/or spleen of free-ranging coyotes from northern California, U.S. Partial DNA sequencing showed that aortic valves from 8 (53%) of 15 coyotes were *B. vinsonii* subsp. *berkhoffii* positive, *B. rochalimae* DNA was amplified from the spleen of one coyote, and *B. henselae* DNA was amplified from the mitral valve of another coyote. By sequence analyses, four coyotes were infected with *B. vinsonii* subsp. *berkhoffii* genotype I, three with genotype II, and one with genotype III (87).

Two species of *Bartonella*, a novel *Bartonella clarridgeiae*-like bacterium and *B. vinsonii* subsp. *berkhoffii*, were isolated from rural dogs and gray foxes in northern California (58). Two *B. henselae* sequences detected in the spleen of raccoon dogs in Korea matched the strain Houston-1 and by ITS sequences were 99.8% similar to a strain found in dogs in China (94). Northern and Southern sea otters were found IFA positive for *B. washoensis* (107, 108). The authors also detected *B. bacilliformis* by PCR in heart valves of both species. A strain close to *B. washoensis* was detected by PCR in Japanese marten (95). Chinnadurai et al. (109) detected a novel strain in river otters by PCR with a sequence matched a strain previously described in Southern flying squirrel. In the Netherlands, harbor seals were found to carry a strain 97% similar to *B. grahamii* (112).

## *Brucella* Species Identified in Wild Carnivores

There are no reports on identification of *Brucella* species by culture or by sequence analysis in animals belonging to Felidae, Herpestidae, and Hyaenidae families. Except for one bobcat that had antibodies against *Br. canis* (115), the other few seropositive Feliformia animals had antibodies against *Br. abortus* (116, 132). Since the authors did not use specific tests that identify rough *Brucella* species, they were not able to find antibodies.

In contrast, multiple *Brucella* species can infect Caniformia animals. *Brucella* species identified by culture or PCR/sequencing in terrestrial carnivores included *Br. canis* in coyotes (137); *Br. abortus* in wolves, red foxes, gray foxes, pampas gray foxes, and raccoons (123, 126, 154, 177); *Br. suis* biovar 4 in wolves, arctic foxes, and red foxes (11, 157, 164); *Br. microti* and *Br. vulpis* in red foxes (159, 161). One red fox species, *V. vulpes*, can carry four different *Brucella* species (*Br. abortus, Br. vulpis, Br. microti*, and *Br. canis*) (115, 125, 159–161). All isolates obtained from arctic foxes were identified as *Br. suis* biovar 4 (11, 13, 48). This is not surprising as reindeer are common hosts of *Br. suis* biovar 4, and arctic foxes often scavenge dead reindeer.

Various aquatic carnivores carry a different species, *Brucella pinnipedialis*. It was identified in the harbor seal (*Phoca vitulina*), the ringed seal (*P. hispida*), the harp seal (*Pagophilus groenlandicus*), the gray seal (*Halichoerus grypus*), the hooded seal (*Cystophora cristata*), Asian sea otter (*Enhydra lutris*), and European river otter (*Lutra lutra*) (168, 171, 207, 208, 214). Characterization of the isolates belonging to this species indicated that *Br. pinipedialis* may contain different biovars (208).

## *Bartonella* and *Brucella* Infections in Wild Carnivores by Family

### Family Felidae

#### Genus *Panthera*

*Bartonella* infection was reported in three big cats species: African lion (*P. leo*), jaguar (*P. onca*), and Far Eastern leopard (*P. pardus orientalis*). *B. henselae* and *B. koehlerae* subsp. *koehlerae* were cultured from the blood of three (5.2%) of 58 lions from Kruger National Park in South Africa (61, 62). The level of bacteremia in the culture-positive lions varied from 35 to 2,000 bacteria per 1 ml of blood. *Bartonella* culture- and antibody-positive lions were found among semi-captive lions from three ranches in South Africa (71). Interestingly, all studied lions from Zambia and Zimbabwe were negative for *Bartonella* by culture and PCR (63, 70). A wild-caught jaguar in Brazil, which was maintained in captivity for only a week, was found *B. henselae* positive (236). This finding led the authors to believe that the animal had been infected in the wild. In the Russian Far East, wild Amur tigers (*P. tigris altaica*) tested negative for antibodies to *B. henselae* (72, 73), but two of five Far Eastern leopards from that area had antibodies against *B. henselae* (73).

Limited information exists about *Brucella* in the wild cats of the genus *Panthera*. During the investigation of *Brucella* infection in the human, livestock and wildlife interface in the Katavi-Rukwa ecosystem in Tanzania, Assenga et al. (130) found one of the two tested lions serologically positive for *Brucella* at a titer 1:200 by three different tests (RBPT, BAPA, and Riv.T). In a 1968 study in Tanzania, Sachs et al. (116) found two of 13 lions had antibodies to *Brucella* species by tube agglutination test. De Vos and Van Niekerk (131) were not able to detect *Brucella* antibodies in four lions from the Kruger National Park, South Africa. Furtado et al. (132) tested serum samples from 31 free-ranging jaguars (*P. onca*) from Brazil using *Br. abortus* as antigen and reported antibodies in one jaguar.

#### Genus *Puma*

Two *Bartonella* species were cultured from mountain lions (*P. concolor*) (55). *Bartonella* antibodies were found in mountain lions from Arizona, California, Idaho, Oregon, Texas, Wyoming, and Florida in the U.S. (17, 68, 69, 78, 79). No *Bartonella* DNA was detected in spleen samples of three mountain lions from Colorado, U.S. (77). *B. henselae* antibodies were found in pumas from Bolivia, Peru, and Venezuela (17). Filoni et al. (60) reported 16 out of 18 pumas serologically positive to *B. henselae* in Brazil. *B. henselae* DNA was detected in lung tissues of three Florida pumas with the first and only up to date reported association of *B. henselae* infection with a fatal disease syndrome of necrotizing interstitial pneumonia and suppurative myocarditis in pumas (76).

All 24 free-ranging Florida panthers (*P. c. coryi*) were seronegative for *Brucella* (134). Reports of *Brucella* in populations of pumas from elsewhere in the Americas were unavailable.

#### Genus *Acinonyx*

The cheetah (*A. jubatus*) is only member of its genus. Kelly et al. (63) reported isolation of *B. henselae* genotype II from an African pet cheetah from Zimbabwe. In 2016, Molia and colleagues (61) isolated *Bartonella* bacteria from blood of 5.9% (1/17) Namibian cheetahs, and the cheetah was infected with a previously unidentified *Bartonella* strain. The Namibian cheetah strain was close but distinct from isolates from North American wild felids and clustered between *B. henselae* and *B. koehlerae;* it was claimed to be a new subspecies of *B. koehlerae* (61). The same study documented that 23% of the 73 animals were positive for *Bartonella* DNA by PCR and 31% (23/74) of cheetahs had antibodies to *B. henselae*. No reports on *Brucella* infections in the cheetah were found.

#### Genus *Lynx*

Those are medium-sized cats represented by four species: Canadian lynx (*Lynx canadensis*), Eurasian lynx (*L. lynx*), Iberian lynx (*L. pardinus*), and bobcat (*L. rufus*). Chomel et al. (55) isolated two *Bartonella* species (*B. henselae* and *B. koehlerae* subsp. *bothieri*) from bobcats in California, U.S. A high prevalence of *Bartonella* antibodies (22.4–74.0%) was reported in bobcats from California, Colorado, Florida, Nevada, and Oregon in the U.S. and from Mexico (17, 56, 68, 69). In northwestern Mexico, a *Bartonella* genotype was found in a flea *P. simulans* collected from a bobcat, but not in the blood of that animal (67). *B. henselae* DNA was found in 16 of 75 (21.3%) blood samples of Iberian lynx from southern Spain (57).

Antibodies against *Brucella* species in bobcats were reported in two studies: *Br. abortus* at 6.6% (5/75) in California (127) and *Br. canis* at 33% (1/3) in Texas (115). Serological investigations of bobcats from Alabama, Texas, and Utah in the U.S. did not result in identification of antibodies to *Brucella* (126, 128, 129). Tessaro (125) reported the absence of *Brucella* bacteria and anibodies in Canadian lynx.

#### Genus *Leopardus*

These are small spotted cats mostly native to Middle and South America. Representatives are the ocelot (*L. pardalis*), the little spotted cat (*L. tigrinus)*, Geoffroy's cat (*L. geoffroyi*), and the margay *(L. wiedii*). Antibodies to *B. henselae* were reported in the ocelot (1/1) and the little spotted cat (2/2) in Brazil (60). A *Bartonella* sequence similar to *B. koehlerae* and *B. henselae* was detected in the captive margay in Brazil (235). The animal was born in the wild and lived in captivity prior to sampling, thus it is not possible to ascertain if the infection was acquired in the wild or in captivity. The authors claimed this animal exhibited clinical signs of bartonellosis: episodes of accentuated weight loss, dullness, dehydration, and anemia (235). The main reason why we have included the case of captive margay into our review is that the identified strain was different from all strains described in domestic and wild felines.

#### Genus *Prionailurus*

This is a genus of small spotted wild cats native to Asia. The genus includes the Iriomote cat (*P. iriomotensis*) and the Tsushima leopard cat (*P. bengalensis euptilura*), both endangered in Japan. A molecular epidemiologic survey in Japan resulted in identification of *B. henselae* in 6% (2/33) of Iriomote leopard cats and *B. clarridgeiae* in 8% (1/13) of Tsushima leopard cats (75). In the following study, four ixodid ticks collected from Tsushima leopard cats were PCR positive for *B. henselae* (74).

#### Genus *Felis*

The European wildcat (*F. silvestris silvestris*) is a subspecies of the same species that includes domestic cats (*F. s. catus*). This species is found in forest habitats of Europe. There are two reports of the presence of *Bartonella* in wildcats from Spain. First, Márquez et al. (65) identified *B. alsatica*, strain associated with rabbits, in a flea *Spilopsyllus cuniculi* collected from a wildcat in Spain. Then, Gerrikagoitia et al. (64) detected *B. henselae* DNA in a carcass of a wildcat. A study of feral cats in the U.S. state of Georgia by Hwang and Gottdenker (59) also reported that 48% of feral cats were PCR positive for three *Bartonella* species: *B*. *henselae, B. koehlerae*, and *B. clarridgeiae*.

### Family Viverridae

The most common species are civets and genets widely distributed in South and Southeast Asia, Africa, and Southern Europe. The first evidence suggesting that civets can host *Bartonella* came from a description of a human cat scratch disease case reported in 2001 in Japan. In the case, a patient scratched by a masked palm civet (*Paguma larvata*) developed fever and inguinal lymphadenopathy with a high antibody titer (1:1,024) to *B. henselae* (237). Later, Sato et al. (82) cultured *B. henselae* from blood of one of 50 masked palm civets collected in Chiba Prefecture of Japan. The level of bacteremia was high (7,000 bacteria per 1 mL of blood). Importantly, the multi-locus sequence type detected from the isolated strain revealed a unique genotype. Though the prevalence of *Bartonella* in cats in Chiba prefecture was 5%, the same genotype had never been found in any *B. henselae* strains from cats from the same and other prefectures (82). *Bartonella* DNA was detected in another Viverridae species, the common genet (*Genetta genetta*). Conducting molecular detection of vector-borne pathogens in wild carnivores in natural parks and adjacent residential areas in Barcelona, Spain, Millán et al. (83) identified *B. clarridgeiae* in tissues of two of 34 (6%) common genets, but ticks collected from genets were free of *Bartonella* DNA. In another study conducted in Northern Spain (Basque County), Gerrikagoitia et al. (64) did not detect *Bartonella* DNA in 13 common genets tested. Márquez et al. (65) also found no *Bartonella* DNA in 18 fleas *S. cuniculi* collected from 10 common genets in Andalusia, Spain.

Reports of *Brucella* testing among viverrids are nearly nonexistent. No *Brucella* antibodies were found in two common genets (*G. genetta*) and three Cape genets (*G. tigrina*) from eastern Africa tested by tube agglutination test (116, 136).

### Family Herpestidae

Mongooses is the common name for the weasel-like small carnivores that live in southern Asia, Africa, and southern Europe, and are introduced to some other areas. We have information about *Bartonella* in one species of this genus—the small Asian mongoose (*Herpestes javanicus*). Sato et al. (82) isolated *B. henselae* from 15.9% (10/63) of small Asian mongooses from Okinawa prefecture, Japan. Based on multi-locus sequence analysis, they identified four types of *B. henselae* strains cultured from mongooses (82). Jaffe et al. (81) tested small Asian mongooses in Grenada and found 32% (54/167) of the animals IFA positive and 35% (18/51) PCR positive for *B. henselae*. The only additional report of investigation of mongooses was from testing a single Egyptian mongoose (*Herpestes ichneumon)* in Algeria and the PCR test was negative (80).

There is a report of antibodies against *Br. abortus* in one white-tailed mongoose (*Ichneumia albicauda*) (33%, 1/3) and one banded mongoose (*Mungos mungo*) (100%, 1/1) in Tanzania (116).

### Family Hyaenidae

The family contains four species of hyenas and phylogenetically belongs to the suborder *Feliformia* despite the dog-like appearance of these animals. The only available report on testing hyenas for *Bartonella* is from a molecular survey of 19 spotted hyenas (*Crocuta crocuta*) from two sites in Zambia with no positive results (70).

Serological observation of 15 spotted hyenas from Tanzania resulted in detection of antibodies against *Brucella* in four out of 15 (27%) animals (116). In the prior study, Sachs and Staak (135) found *Brucella* species exposure in two out of four hyenas in Tanzania. Another serological study did not detect *Brucella* antibodies in two spotted hyenas from the Kruger National Park in South Africa (131).

### Family Canidae

#### Genus *Canis*

Multiple wild species, including coyotes, jackals, and wolves belong to this genus. The golden jackal (*C. aureus*) is a species experiencing rapid geographic expansion with significant public health impacts (238). Of 57 golden jackals sampled from four sites in Iraq, seven (12.3%) were PCR positive for *Candidatus* B. merieuxii and one (2%) for *B. vinsonii* subsp. *berkhoffii* (86). In Israel, Marciano et al. (85) found nine out of 70 (13%) golden jackals PCR positive for *Bartonella* species: 5/9 *B. rochalimae*, 3/9 close to *Candidatus* B. merieuxii, and 1/9 between *B. vinsonii* subsp. *berkhoffii* and *Candidatus* B. merieuxii. A search for *Bartonella* in coyotes (*C. latrans*) from California and Colorado in the U.S. and from Mexico demonstrated a high prevalence of up to 89% by IFA, 42% by culture, and 28% by PCR (58, 67, 77, 87–91). There is one report of PCR detection of *Bartonella* DNA in a wolf (*C. lupus*) from northern Spain (64).

Most reports of *Brucella* infections in canids are based on detection of antibodies. Serologically positive coyotes were identified from California and Texas, U.S. (115, 117, 127, 128, 142). In wolves, evidence of *Brucella* infections also included *Brucella* isolations in Canada and Russia (13, 125). *Brucella* was found in two jackal species: 1.9% (4/216) of golden jackals (*C. aureus*) in Serbia were positive for *Br. canis* by PCR (137) and 43% (3/7) of black-backed jackals (*C. mesomelas*) in Tanzania were seropositive for *Br. abortus* by tube agglutination test (116).

#### Genus *Vulpes*

There are more reports on detection of *Bartonella* in red foxes (*V. vulpes*) than in any other species of wild carnivores. *Bartonella* DNA was identified in red fox tissues from Australia, Austria, France, Israel, Spain, and U.S. (64, 77, 83, 85, 88, 103, 105). Most sequences were identified as *B. vinsonii* subsp. *berkhoffii* and *B. rochalimae*. Out of two red foxes positive for *Bartonella* DNA in Israel, one harbored DNA sequences that were 100% identical to *B. rochalimae* and the other was positive for *Candidatus* B. merieuxii (85). Hodžić et al. (104) did not detect *Bartonella* DNA in 119 fox spleen samples from Bosnia and Herzegovina. Blood samples from 39 red foxes from Iraq were also negative for *Bartonella* by PCR; however, 12.8% of these foxes were serologically positive for *Bartonella* antibodies (86). Mascarelli et al. (100) detected *B. henselae* DNA in three out of 20 tested arctic foxes (*V. lagopus*) from Canada and López-Pérez et al. (67) identified *B. rochalimae* DNA in two out of 15 kit foxes (*V. macrotis*) tested.

There are several reports about screening of ectoparasites from red foxes. DNA of a *Bartonella* strain, closely related to *B. rochalimae*, was found in fleas (*Pulex irritans*) from red foxes in Andalusia, Spain (65). PCR tests detected *B. clarridgeiae* and *B. henselae* in 20/34 and 4/34 fleas (*Ctenocephalides felis*), respectively, from red foxes in Australia, where it is an introduced species (105). Sréter-Lancz et al. (106) found *Bartonella* DNA in 4.2% (4/95) pools of fleas (*P. irritans*) from red foxes in Hungary, but all ticks from foxes were negative.

Similarly, there are numerous reports of *Brucella* infections in red foxes in Austria, Canada, Ireland, Russia, the U.S., and the UK (115, 125, 159–161). Tessaro (125) cultured *Br. abortus* from red foxes in Canada. Morton (48) cultured *Br. suis* biovar 4 from three out of 38 red foxes from Alaska. Scholtz et al. (161) cultured *Br. microti* and the proposed novel species *Br. vulpis* from red foxes in Austria in 2016. *Br. suis* biovar 4 cultures were obtained from arctic foxes from Alaska and Russia (11, 13, 48). McCue and O'Farrell (158) conducted a serological survey of San Joaquin kit foxes in California, U.S. and reported antibodies to *Br. abortus* in 8% in 1981–1982 and 3% in 1984 and to *Br. canis* in 14% in 1981–1982 and none in 1984.

#### Genus *Cerdocyon*

Investigators tested another fox species, the crab-eating fox (*Cerdocyon thous*), in Brazil and found *B. rochalimae* DNA in all nine *P. irritans* fleas collected from one animal (92). In another study by De Sousa et al. (66), none of the 78 sampled crab-eating foxes showed presence of *Bartonella* DNA in blood samples by qPCR.

#### Genus *Urocyon*

This genus contains two species of Western Hemisphere foxes: the gray fox (*U. cinereoargenteus*) and closely related island fox (*U. littoralis*), which is a dwarf cousin of the gray fox (239). There is a comprehensive study of *Bartonella* in gray foxes in northern California, U.S., conducted by Henn et al. (58). A novel *B. clarridgeiae*-like bacterium was isolated from 22 (42%) of 53 gray foxes and *B. vinsonii* subsp. *berkhoffii* from five gray foxes (9.4%). Serology showed that 48 gray foxes (89%) had detectable antibodies against *Bartonella*. The authors made the conclusion that the high prevalence of bacteremia and seroreactivity in gray foxes suggests that they may act as a reservoir species for the *B. clarridgeiae*-like species in this region. In another study of gray foxes in northern California, 14 (64%) of 22 foxes were infected with *Bartonella* species at one or more of the capture dates (97). Fleas collected from gray foxes in the study were identified as *P. simulans*, and 39% of the fleas were PCR positive for *Bartonella*, with *B. rochalimae* and *B. vinsonii* subsp. *berkhoffii* identified in 81% and 19% of the PCR positive fleas, respectively.

A serological survey of 132 gray foxes from Texas, U.S., demonstrated an antibody prevalence of 50% (66/132), with 22 (33.3%) individuals seropositive for *B. clarridgeiae*, eight (12.2%) for *B. vinsonii* subsp. *berkhoffii*, and 36 (54.5%) for both *B. clarridgeiae* and *B. vinsonii* subsp. *berkhoffii* (96). In gray foxes from Colorado, U.S., and northern Mexico *Bartonella* DNA was not detected (67, 77). Serological survey of the endangered island foxes (*U. littoralis*) conducted on several islands near the Californian coast by Namekata et al. (99) demonstrated a wide range of seroprevalence for *B. clarridgeiae* and *B. vinsonii* subsp. *berkhoffii* from 0% on San Nicolas Island to 86% on Santa Cruz Island. The following serological survey of 51 island foxes on Santa Rosa Island identified the overall antibody prevalence of 62.7% with 16 (31.4%) foxes seropositive for *B. clarridgeiae* only, five (9.8%) for *B. vinsonii* subsp. *berkhoffii* only, and 11 (21.6%) for both antigens (98). Importantly, *B. vinsonii* subsp. *berkhoffii* was isolated from six (11.8%) foxes using blood culture medium. All of the isolated *B. vinsonii* subsp. *berkhoffii* belonged to type III, the same type found in mainland gray foxes (98).

A culture of *Br. abortus* was obtained from one gray fox (*U. cinereoargenteus*) from Alabama, U.S. (126) while there were no positive results in foxes of this species in Arkansas, Florida, and South Carolina, U.S. (115, 156).

#### Genus *Lycalopex*

Several investigations of the South American foxes for *Brucella* infection have been published, including those investigating the pampas gray fox (*L. gymnocercus*) and Patagonian gray fox (*L. griseus*). Szyfres and González Tomé (154) found evidence of *Brucella* in both species from Argentina and isolated *B. abortus* biovar 1 from a pampas gray fox.

#### Genus *Nyctereutes*

The DNA identified as *B. henselae* was detected in spleens of two out of 142 raccoon dogs (*N. procyonoides*) in Korea, but not in any of 51 blood samples tested (94).

### Family Ursidae

We found research on *Bartonella* and *Brucella* in three bear species, namely black bear (*U. americanus*), brown bear (*U. arctos*), and polar bear (*U. maritimus*). *Bartonella* DNA was not detected in seven black bears from Colorado, U.S. (77). All other research was focused on *Brucella* in bears (119, 147, 148, 179, 180, 182–185). Despite high seroprevalence levels for *Br. abortus* antibodies in all investigated bear species, we could not find any report on successful isolation of *Brucella* from these animals. Serological tests of 61 black bears for *Br. canis* by Bronson et al. (178) were negative.

### Family Mephitidae

Twelve skunks of two species, the hooded skunk (*Mephitis macroura*) and the striped skunk (*M. mephitis*), from Colorado, U.S., and Mexico were found infected with *B. rochalimae* and one skunk from Mexico was infected with *B. vinsonii* subsp. *berkhoffii* (67, 77).

Antibodies against *B. abortus* were found in 8.7% of striped skunks and 3.9% of western spotted skunks (*Spilogale gracilis*) from California, U.S. (127).

### Family Procyonidae

The common raccoon (*Procyon lotor*) has a natural range from southern Canada to Panama. Of 37 raccoons trapped on St. Simon Island in Georgia, U.S., 12 were positive for *B. henselae* and one for *B. koehlerae* (59). Interestingly, raccoons from the western regions of the U.S. carried different species of *Bartonella*. Henn et al. (88) isolated *B. rochalimae* from 11 of 42 raccoons from California, and Bai et al. (77) found 11 of 186 raccoons from Colorado PCR positive for *B. rochalimae* and three for *B. vinsinii* subsp. *berkhoffii*. All 977 raccoons from Japan, where it is an introduced species, were PCR negative for *Bartonella* (95).

Two *Brucella* strains cultured from raccoons from Alabama were identified as *Br. abortus* biovar 1 (126, 177). Raccoons seropositive to *Brucella* species were found in California, Alabama, Florida, and Texas in the U.S. (115, 126–128). None of 63 raccoons from Nebraska, U.S., had antibodies to *Br. canis* (139). In South Korea, *Brucella* DNA was found in blood (1/9) and tissues (2/5) of introduced raccoons (172). Three (8.8%) of 34 brown-nosed coatis (*Nasua nasua*), which also belong to family Procyonidae, were serologically positive for *Brucella* in the Brazilian Pantanal (149).

### Family Mustelidae

Mustelidae is the largest family in the order Carnivora. Many terrestrial species of this genus were tested for *Bartonella*, including the beech marten (*Martes foina*), pine marten (*M. martes*), Japanese marten (*M. melampus*), American badger (*Taxidea taxus*), stoat (*Mustela erminea*), Japanese weasel (*M. itatsi*), least weasel (*M. nivalis*), Siberian weasel (*M. sibirica*), American mink (*M. vison*), European polecat (*M. putorius*), and ferret (*M. putorius furo*) (Table 1). However, out of 16 mustelid species tested for *Bartonella* DNA, only two cultures were obtained: one from a Japanese badger (*Meles anakuma*) and another from a Japanese marten (95). The isolate from the marten was close to *Bartonella washoensis*, a species typically found in squirrels, suggesting that it could have potentially “jumped” from a squirrel to its natural predator. The isolate from the Japanese badger was unique, with the closest match being to *B. clarridgeiae* and *B. rochalimae* (95). *Bartonella clarridgeiae* or related sequences were also detected in a beech marten and in European badgers, all from Spain (64, 83).

In North Carolina, U.S., Chinnadurai et al. (109) revealed a novel *Bartonella* species in 19 (29%) of 65 tested river otters (*Lontra canadensis*). *Bartonella* infection was detected in 45% (23/51) and 10% (3/30) of heart valves of northern and southern sea otters (*Enhydra lutris kenyoni* and *E. l. nereis*), respectively, by PCR (108). Analysis of the *Bartonella* ITS region identified two *Bartonella* species in those animals: a novel species closely related to *Bartonella washoensis* and *Candidatus* B. volans, whereas another genotype was molecularly identical to *B. henselae*. Sera from 50% of necropsied and 34% of presumed healthy, live-captured northern sea otters and in 16% of necropsied southern sea otters contained antibodies against *Bartonella* species (107).

Antibodies against *Brucella* species were detected in the American badger (*Taxidea taxus*), American mink (*Neovison vison*), European mink (*Mustela lutreola*), Eurasian otter (*Lutra lutra*), wolverine *(Gulo gulo)*, and northern, southern and Asian sea otters (*Enhydra lutris keyoni, lutris, nereis*) from Europe, Asia, North and South Americas (11, 124, 125, 127, 174). We found only one report of successful culturing of *Brucella* (*Br. abortus*) from terrestrial mustelids (farmed European mink) and only one report of PCR detection of *Brucella* DNA in tissues of Asian badger (*Meles leucurus*) (172, 240). Similar to *Bartonella*, sea otters carry different species of *Brucella* than terrestrial mustelids. Investigating rectal swab samples of Asian sea otters (*E. l. lutris)* from Russia (168) found DNA of three *Brucella* species (*Br. abortus, Br. melitensis*, and *Br. pinnipedialis*). Miller et al. (169) isolated marine *Brucella* from a southern sea otter (*E. l. nereis*) with osteolytic lesions that was stranded on the central California coast. Antibodies to *Brucella* were detected in Northern sea otters (*E. l. keyoni*) from Alaska in the U.S. and Russia (120).

### Families Phocidae, Otariidae, and Odobenodae

There is only one report on the identification of *Bartonella* in any of the pinnipeds, including walruses, eared seals, and true seals. Morick et al. (112) tested spleen samples and seal lice (*Echinophtirius horridus*) collected from seven harbor seals (*Phoca vitulina*). One spleen of 48 tissue samples and one of six lice pools were positive. The *Bartonella* species identified in the spleen and lice were found to be identical to each other by two genetic loci. One genetic marker identified the genotype as *B. henselae*, while another marker indicated 97% sequence similarity with *B. grahamii*.

In contrast to *Bartonella*, there is abundant evidence of *Brucella* infections in various species of the clade Pinnipedia. In family Phocidae (true seals), cultures of *Brucella* species were obtained from hooded seals (*Cystophora cristata)*, gray seals (*Halichoerus grypus*), ringed seal *(Phoca hispida)*, harp seal (*Pagophilus groenlandicus*), and harbor seal *(Ph. vitulina)* (170, 171, 206, 208, 214, 223, 224, 226, 231, 233). All identified cultures from true seals were *Br. pinnipedialis*. Serological evidence of *Brucella* was reported from investigation of even more species of true seals, including the bearded seal (*Erignathus barbatus*), ribbon seal (*Histriophoca fasciata)*, leopard seal (*Hydrurga leptonyx*), Weddell seal (*Leptonychotes weddellii*), crab-eater seal (*Lobodon carcinophaga*), southern elephant seal (*Mirounga leonina*), Hawaiian monk seal (*Neomonachus schauinslandi*), Ross seal (*Ommatophoca rossii*), and several species of the genus *Phoca*.

In the family Odobenidae (walruses), Nielsen et al. reported serological prevalence of 12% (7/59) in 1996 and 3% (5/170) in 2001 in Atlantic walrus (*Odobenus rosmarus rosmarus*) from Canada; however serological tests of 40 Pacific walruses (*O. r. divergens*) from Alaska by Calle et al. (211) showed no antibodies to *Brucella* species

There are multiple reports of *Brucella* antibodies in fur seals and sea lions of the family Otariidae—nine species of the genera (*Arctocephalus, Callorhinus, Eumetopias, Neophoca, Phocarctos*, and *Zalophus*) (Table 2).

## Differences in Distribution of *Bartonella* and *Brucella* Species in Wild Carnivores

Carnivores have regular exposure to both *Bartonella* and *Brucella* bacteria through predation on pathogen hosts, scavenging, and arthropod vectors. As with plague caused by *Yersinia pestis* (241), testing one carnivore for *Bartonella* and *Brucella* species could be equivalent to sampling a large number of its prey animals and give an idea of the epidemiological situation in the local environment. Overall, both *Bartonella* and *Brucella* are common in wildlife. Our review demonstrated numerous reports of infections caused by bacteria of both taxa in wild carnivores. We analyzed over 170 *Bartonella* and *Brucella* studies covering 109 species and subspecies of carnivores (Table 3). Eighty-four species of carnivores were tested for *Brucella* and 79% of these species were found positive by serological, bacteriological, or molecular methods. Out of 51 species examined in *Bartonella* studies, 71% tested positive.

**Table 3:** *Bartonella* and *Brucella* studies in wild carnivores by species.

**Host species**	**+/−**	***Bartonella* ref**.	***Brucella* ref**.	**+/−**
**SUBORDER FELIFORMIA**				
**Family Felidae**				
Cheetah (*Acinonyx jubatus*)	+	(61–63)		
Wildcat *Felis silvestris*)	+	(64, 65)		
			(124)	−
Ocelot (*Leopardus pardalis)*	+	(60)		
	−	(66)		
Little spotted cat (*Leopardus tigrinus)*	+	(60)		
Iberian lynx	+	(57)		
(*Lynx pardinus)*	−	(65)		
Lynx (*Lynx canadensis*)			(125)	−
Bobcat *(Lynx rufus)*	+	(17, 55, 56, 67–69)	(115, 127)	+
			(126, 128, 129)	−
African lion *(Panthera leo*)	+	(61, 62, 71)	(116, 130)	+
	−	(63, 70)	(131)	−
Jaguar (*Panthera onca*)			(132)	+
			(133)	−
Leopard (*Panthera pardus*)			(116)	−
Far Eastern leopard	+	(73)		
(*Panthera pardus orientalis*)	−	(72)		
Amur tiger (*Panthera tigris altaica*)	−	(72, 73)		
Iriomote cat (*Prionailurus bengalensis*	+	(75)		
*iriomotensis)*	−	(74)		
Tsushima leopard cat (*Prionailurus bengalensis)*	+	(74, 75)		
Mountain lion (*Puma concolor*)	+	(17, 55, 60, 68, 69, 76, 78, 79)		
	−	(77)	(134)	−
**Family Herpestidae**				
Small Indian mongoose *(Herpestes javanicus)*	+	(81, 82)		
Egyptian mongoose *(Herpeses ichneumon)*	−	(80)		
White-tailed mongoose (*Ichneumia albicauda*)			(116)	+
Banded mongoose (*Mungos mungo*)			(116)	+
**Family Hyaenidae**				
Spotted hyena (*Crocuta crocuta*)			(116, 135)	+
	−	(70)	(131)	−
**Family Viverridae**				
Common genet (*Genetta genetta*)	+	(83)		
	−	(64, 169)	(136)	−
Cape genet (*Genetta tigrina*)			(116)	−
Masked palm civet *(Paguma larvata)*	+	(82)		
**SUBORDER CANIFORMIA**				
**Family Candiae**				
Golden jackal *(Canis aureus)*	+	(85, 86)	(137)	+
	−	(80, 84)		
Coyote *(Canis latrans)*	+	(58, 67, 77, 87–91)	(115, 117, 127, 128, 142)	+
			(126, 129, 138–141, 143)	−
Wolf (*Canis lupus)*	+	(64)	(11, 13, 53, 118, 125, 144–146, 148)	+
			(115, 124, 147)	−
Black-backed jackal (*Canis mesomelas*)			(116)	+
Crab-eating fox (*Cerdocyon thous*)	+	(92)	(149)	+
	−	(66)	(150–152)	−
Maned wolf (*Chrysocyon brachyurus)*			(150)	−
Patagonian gray fox (*Dusicyon griseus griseus)*			(153, 154)	+
Pampas gray fox (*Dusicyon gumnocercus antiquus*)			(153, 154)	+
Darwin's fox (*Lycalopex fulvipes*)	−	(93)		
Pampas fox (*Lycalopex gymnocercus)*			(151)	−
Hoary fox (*Lycalopex vetulus)*			(155)	+
Wild dog (*Lycalopex pictus)*			(116)	+
	−	(70)		
Raccoon dog	+	(94)		
(*Nyctereutes procyonoides)*	−	(95)		
Bat-eared fox (*Otocyon megalotis)*			(116)	−
Gray fox *(Urocyon cinereoargenteus)*	+	(58, 67, 96, 97)	(126)	+
	−	(77)	(115, 156)	−
Island fox *(Urocyon littoralis)*	+	(98, 99)		
Arctic fox (*Vulpes lagopes*)	+	(100)	(11, 13, 48, 53, 124, 157)	+
Kit fox *(Vulpes microtis)*	+	(47)		
			(129)	−
San Joaquin kit fox			(158)	+
(*Vulpes macrotis mutica)*			(152)	−
Red fox (*Vulpes vulpes*)	+	(64, 65, 77, 83, 85, 86, 88, 103, 105, 106)	(48, 115, 124, 125, 146, 148, 159–164)	+
	−	(102, 104)	(156)	−
**Family Mephitidae**				
Hooded skunk (*Mephitis macroura*)	+	(67)		
Striped skunk *(Mephitis mephitis)*	+	(67, 77)	(127)	+
			(115, 128, 156)	−
Western spotted skunk (*Spilogale gracilis*)			(127)	+
**Family Mustelidae**				
Northern sea otter (*Enhydra lutris keyoni*)	+	(107, 108)	(120, 165, 167)	+
			(166)	−
Asian sea otter *(Enhydra lutris lutris)*			(120, 168)	+
Southern sea otter (*Enhydra lutris nereis*)	+	(107, 108)	(167, 169)	+
Steppe polecat (*Mustela eversmanii)*			(124)	−
Japanese weasel *(Mustela itatsi)*	−	(95)		
European mink (*Mustela lutreola)*			(124)	+
Least weasel *(Mustela nivalis)*	−	(64, 110)	(173)	−
European polecat (*Mustela putorius*)	−	(64)		
Ferret (*Mustela putorius furo*)	−	(110)		
Siberian weasel *(Mustela sibirica)*	−	(95)		
American mink *(Mustela vison)*			(174)	+
	−	(64)		
Fisher (*Pekania pennant*)			(125)	−
American badger (*Taxidea taxus*)	+	(67, 111)	(127, 128)	+
			(129, 175)	−
**Family Procyonidae**				
Brown-nosed coati *(Nasua nasua)*			(149)	+
	−	(66)		
Raccoon *(Procyon lotor)*	+	(59, 77, 88)	(115, 126–128, 172, 177)	+
	−	(67, 95)	(139, 156, 176)	−
**Family Ursidae**				
Black bear (*Ursus americanus*)			(147, 179, 180)	+
	−	(77)	(178)	−
Brown bear *(Ursus arctos)*			(179)	+
Alaska peninsula brown bear *(Ursus arctos gyas)*			(119)	+
Grizzly bear *(Ursus arctos horriblis)*			(48, 119, 144, 148, 181)	+
Kodiak brown bear *(Ursus arctos middendorffi)*			(119)	+
Marsican brown bear (*Ursus arctos marsicanus)*			(182)	+
Polar bear (*Ursus maritimus)*			(179, 183–185)	+
**SUPERFAMILY PINNIPEDIA**				
**Family Odobenidae *(Walruses)***				
Pacific walrus (*Odobenus rosmarus divergens*)			(186)	−
*Atlantic walrus (Odobenus rosmarus rosmarus)*			(187, 188)	+
**Family Otariidae *(fur seals and sea lions)***				
South American fur seal (*Arctocephalus australis*)			(189)	+
New Zealand fur seal *(Arctocephalus forsteri)*			(190)	−
Antarctic fur seal (*Arctocephalus gazella*)			(193–195)	+
			(191, 192)	−
Australian fur seal (*Arctocephalus pusillus doriferus*)			(122, 196, 197)	+
Guadalupe fur seal (*Arctocephalus townsendi)*			(198)	−
Northern fur seal *(Callorhinus ursinus)*			(200)	+
			(199)	−
Steller sea lion (*Eumetopias jubatus*)			(199, 201)	+
Western Steller's sea lion (*Eumetopias jubatus jubatus*)			(202)	+
Australian sea lion (*Neophoca cinerea*)			(197)	+
New Zealand sea lion (*Phocarctos hookeri*)			(203)	+
California sea lion (*Zalophus californianus)*			(121, 204)	+
**Family Phocidae (True seals)**				
Hooded seal *(Cystophora cristata)*			(170, 171, 187, 206–209)	+
			(205)	−
Bearded seal			(210)	+
(*Erignathus barbatus)*			(209, 211)	−
Ribbon seal (*Histriophoca fasciata)*			(199, 202)	+
Gray seal (*Halichoerus grypus*)			(170, 171, 187, 208, 212–216)	+
Leopard seal (*Hydrurga leptonyx*)			(197)	+
Weddell seal (*Leptonychotes weddellii)*			(191, 192, 195, 217, 219)	+
			(218)	−
Crab-eater seal (*Lobodon carcinophaga*)			(192)	+
Southern elephant seal (*Mirounga leonina*)			(191)	+
			(193)	−
Hawaiian monk seal (*Neomonachus schauinslandi)*			(221, 222)	+
			(220)	−
Ross seal (*Ommatophoca rossii*)			(192)	+
Harp seal (*Pagophilus groenlandicus*)			(170, 187, 209, 223, 224)	+
Ringed seal *(Phoca hispida)*			(187, 188, 199, 205, 209, 224)	+
			(170, 207)	−
Spotted seal (*Phoca largha)*			(199, 202)	+
Baikal seal (*Phoca sibirica*)			(216)	−
Harbor seal *(Phoca vitulina)*	+	(112)	(169–171, 187, 199, 208, 212, 214–216, 223, 225, 226, 228–230)	+
Western Pacific harbor seal *(Phoca vitulina stejnegeri)*			(202)	+
Pacific harbor seal *(Phoca vitulina richardsi)*			(231–233)	+

Although no species of wild carnivores were tested for both pathogens in a single study, 26 species were tested for both pathogens in different studies. Of those, 15 (58%) species were positive for both *Bartonella* and *Brucella* (among them bobcat, African lion, golden jackal, coyote, wolf, foxes, striped skunk, sea otters, raccoon, and harbor seal), meaning these carnivores can harbor either pathogen or potentially both. We know that other mammalian groups [bats for example, (242)] can be co-infected with *Bartonella* and *Brucella* species, and we speculate that this is also possible in carnivores, a hypothesis that definitely needs more investigation.

The most commonly identified *Bartonella* species was *B. henselae*, which was found in at least 23 species of wild carnivores, followed by *B. rochalimae* in 12, *B. clarridgeiae* in ten, and *B. vinsinii* subsp. *berkhofii* in seven species. Similarly, *Br. abortus* led the list of *Brucella* species, being identified in 36 terrestrial carnivore species, followed by *Br. canis* in eight. However, most of the reports are based on serology that cannot reliably discriminate these species until there are bacteriological data or sequences of PCR amplicons. *Br. pinnipedialis* is prevalent in marine carnivores, and some of the early reports of antibodies to *Br. abortus* in marine animals probably can be attributed to *Br. pinnipedialis* as well.

The analysis revealed some striking differences in distributions of these infectious agents in wild populations belonging to different carnivore families. One of the evident differences is abundance of several species of *Bartonella* practically in every explored species of wild felids. In contrast, very few reports of *Brucella* in the same species are available and those are limited to detection of antibodies that may indicate an exposure to the agent rather than direct involvement of these animals in the circulation of *Brucella*. At the same time, we could not find any report of *Bartonella* in bears while the presence of *Brucella* in these animals was well documented. An even more evident difference was found in marine carnivores, such as seals and sea lions, with practically every species reported infected with a specific species of *Brucella* (*Br. pinnipedialis*). In contrast, there is only one report of detection of *Bartonella* DNA in one tissue sample of a seal and there is no evidence of a *Bartonella* strain specific to marine mammals. A comparison with other marine mammals, such as dolphins, porpoises, and whales, which were not the subjects of our paper, also indicated a presence of specific *Brucella* species in blood of these animals, known as *Brucella ceti*. Whereas, the cat pathogen *B. henselae* was found in cetaceans, albeit less commonly than species of *Brucella* (243, 244).

Prevalence and the spectrum of bacterial species present depends on a potential exchange of bacteria between domestic and wild terrestrial carnivores. Wild carnivores are often infected with the same pathogens as their domesticated relatives (cats and dogs) though the risk of exposure varies widely because of differences in biology, distribution, and historical interactions. Confirmation of the identity of the bacterial species, however, remains critical for making such a statement regarding host specificity. Using a rapid test for differentiation of *Bartonella* species without sequencing amplicons, Carver et al. (114) came to the conclusion that free-ranging felids (pumas and bobcats) could be infected with *Bartonella* species that are generally considered to cross felid species barriers from domestic cats. Sequence analysis of some cultures and PCR amplicons has challenged such a conclusion. For example, in Californian mountain lions and bobcats Chomel et al. (55) found *Bartonella* species, typical for domestic cats (*B. henselae* and *B. koehlerae*); however, their detailed analysis demonstrated that these strains were sufficiently different for them to propose new subspecies of *B. koehlerae* (55). The authors who described the novel strains noted that these strains appear highly adapted to their particular species of wild cats and likely originated from a common ancestor.

There are some limitations in the analysis provided herein on the distribution of *Bartonella* and *Brucella* species in wild carnivores. The timing of samples collection for the animals listed in our review varied among studies and this factor could influence prevalence of infections. Differences in diagnostic methods used for identification can significantly affect comparison of the results. For a number of reasons, the number of *Brucella* studies relying on detection of antibodies in wild carnivores was much higher compared to the number of *Bartonella* studies in the same species that included either culturing or molecular detection. Several species of *Brucella (Br. suis, Br. abortus*, and *Br. melitensis)* are select agents and culturing of these species requires BSL-3 level capacity. Investigations of *Brucella* in wildlife started much earlier than similar investigations of *Bartonella* when DNA amplification techniques were not available. We should be carefull with interpretation of *Brucella* antibodies since available serological tests cannot identify all species of *Brucella*. There are separate tests for rough *Brucella* species (*Br. canis*) and for smooth *Brucella* species (*Br. abortus, Br. melitensis*, and *Br. suis*), and reported serology depends on the used tests.There are more described species of *Bartonella* (>35) and multiple diverse strains exist within this genus than for *Brucella* species. For many decades, the genus *Brucella* included six species, with some experts arguing that this genus is monospecific. In the past decade, new and more diverse *Brucella* species have been described (7). Recognition of the ubiquitous presence of *Brucella* in the environment will most likely continue (6). Nevertheless, reports of *Brucella* in wildlife without discrimination between species and biovars are still common, whereas future studies of *Bartonella* infections are more likely to be accompanied by proper identification down to species or subspecies level. Clearly, serological investigations are less informative for identification of bacterial species because of possible cross-reactivity between different antigens. The analysis presented in this review demonstrates the need for more information on genetic polymorphism of bacterial pathogens for the purposes of making comparison of strains from domestic and wild carnivores.

## Evolutionary Aspects

Another issue that may influence the choice of methods for discriminating among *Bartonella* species is the effective level of association between these bacteria and their mammalian hosts, ranging from host species to host genus (245). Presumably, such a close bacteria-host association relates to the long-history of co-adaptation between *Bartonella* and their mammalian hosts and possibly arthropod vectors (245). An association of these bacteria with rodents, bats, and ruminants is described elsewhere, but analysis of the literature on *Bartonella* in wild carnivores also supports some degree of host-specificity (e.g., *B. henselae* in felids and *B. vinsonii* subsp. *berkhoffii* in canids).

A co-adaptation of *Brucella* with terrestrial wild carnivore hosts is not as straightforward as in domestic animals. A clear exception to this observation is *Br. pinnipedialis*, a species found in true seals only. Typical for domestic dogs, *Br. canis* may be expected to be commonly shared with wild canids, such as wolves and coyotes. However, this bacterial species has not been cultured from these predators and only few serological findings are available (115, 128, 139). Noticing the absence of *Br. canis* in wolves and coyotes, Moreno (7) proposed that this bacterial species evolved in the dog's ancestor after its predation on *Br. suis* biovar 4 infected animals (e.g., caribou/raindeer). This can be also explained by lack of specific serological tests available and low yield of culture.

Recent phylogenetic reconstructions and diversification analyses of prokaryotes have led to a better understanding of patterns of bacterial macroevolution. According to the analysis of prokaryote evolution based on the 16S rRNA gene (246), the common ancestor between the *Brucella* and the *Bartonella* genera split from the common ancestor with Phyllobacteriaceae in the order Rhizobiales about 567 million years ago and diverged about 507.4 million years ago (247) around the time of the Cambrian explosion and diversification of life during the Paleozoic Era, still on the giant supercontinent Pangea. As the species of the order Rhizobiales most closely related to *Bartonella* and *Brucella* are symbionts on plant roots, we can speculate that the ancestor of the two genera may have been a plant symbiont as well.

*Bartonella* evolved around 134 million years ago during Early Cretaceous Period around the time the flowering plants appeared in the middle of the dinosaur era (247). Segers et al. (248) suggest that the last common ancestor of the *Bartonella* was a gut symbiont of insects that produced its own amino acids and vitamins and that the adaptation to blood-feeding insects facilitated colonization of the mammalian bloodstream. Indeed, Bartonellaceae species were identified in honeybees (248, 249) and ants (250) filling the gap between the pathogenic *Bartonella* clade and more ancient bacterial symbionts. The honeybee strains of *B. apis* form a clade basal to species of the genus *Bartonella* (249). However, the *B. apis* genomes are almost twice as large (2.6 to 2.9 Mb) as the ant symbionts, suggesting that the association with the bee is more recent or that the association is less intimate (251). The phylogenetic trees show that the ant-related bacterial clade is a sister group to bee-related clade and other mammal-related *Bartonella* species (249, 252). Ants predate bees by some 35 million years in the order Hymenoptera which is 325 million years old itself (246). We can only speculate how the *Bartonella* ancestor adapted from a plant symbiont to gut symbiont through possible consumption routes and suggest looking into other “ancient” insect orders, like Archaeognata, or the orders that have maintained connection with water in their metamorphosis, like mayflies or dragonflies; and the ones that include sap-sucking insects.

Genomic and functional similarities between *Br. suis* and organisms from the *Rhizobium Agrobacterium* group suggest that the *Brucella* may have evolved from a soil plant-associated ancestral bacteria and speculatively, it may be metabolically active outside of a mammalian host (253). According to the analysis of prokaryote evolution based on the ribosomal gene, the genus *Brucella* is much younger than *Bartonella* and diverged about 230 thousand years ago (247) during Middle Pleistocene epoch. Previously it was hypothesized that *Brucella* species diverged roughly 20 million years ago following the divergence of their bovine and goat hosts (254). However, whole-genome-based phylogeny (255) supports the ribosomal gene analysis suggesting a much younger age for *Brucella* than previously estimated. Their rooted phylogeny suggests that brucellosis in various mammalian species emerged from infected sheep roughly in the past 86,000 to 296,000 years. This analysis has also suggested that transmittal of *Brucella* from pigs to canids likely happened within the past 22,500 years from infection of wolves or other canids feeding on pigs that were themselves infected (255). So, while possible paleo-brucellosis cases in the Bronze Age and later (256) fit perfectly within the timeframe, the possibility of brucellosis in a 2.5-million-year old hominid (257) brings an exciting prospect of an ancestral *Brucella*-like strain that either became extinct or has not been detected yet.

## Conclusion

We can only speculate that a longer period of evolution of *Bartonella* has resulted in higher diversity and better co-adaptation to specific mammalian hosts compared to *Brucella*. Asymptomatic persistence of *Bartonella* bacteria in their natural reservoir animals contrasts with the well-documented pathological manifestations of *Brucella* in host animals. The only until the present time association of *Bartonella* infection with fatal cases of clinical disease in wild carnivores was reported in Florida pumas (76). Three diseased pumas had spent time in captivity prior to being released in the wild and were found later exhibiting respiratory signs and reluctance to move. Autopsy findings included necrotizing interstitial pneumonia and suppurative myocarditis associated with *B. henselae* infection (76). There is much more information on pathology caused by *Brucella* in domestic animals than in wildlife in general and even less in wild carnivores. Describing a range of pathologies caused by *Brucella* in sea mammals, Foster et al. (208) listed sub-blubber abscesses, hepatic and splenic necrosis, macrophage infiltration in liver and spleen, possible abortion, epididymitis, and meningitis.

In spite of shared mammalian reservoirs, the difference in transmission cycles presents distinct ecological traits. While *Bartonella* species use arthropod vectors as a main mechanism for transmission between mammalian hosts, the role of arthropod vectors in transmission of *Brucella* remains disputed. In our review, we provided some data, mostly from Russian sources, which support a potential role of ticks and other arthropods in transmission of *Brucella*. Nevertheless, it is hard to argue that such means of transmission are significant, let alone dominant, in transmission of these bacteria. Commonly, wild terrestrial predators contract brucellosis through consumption of infective tissues during predation and scavenging (258). Considering potential modes of *Brucella* transmission between marine mammals, Foster et al. (208) also included social interactions, sexual activity, maternal transmission, physical trauma, ingestion during feeding, and carriage by parasites.

We realize that our analyses create more questions than answers; the current review brought up significant parallels and differences in *Bartonella* and *Brucella* ecologies in wild carnivores and we hope it will prove to be useful for a wide range of specialists and can stimulate interest in comparing the ecologies of *Bartonella* and *Brucella* in wildlife and, at a larger scale, in investigating ecological trends of phylogenetically related zoonotic agents; benefitting epidemiological research and wildlife conservation.

## Author Contributions

All authors listed have made a substantial, direct and intellectual contribution to the work, and approved it for publication.

### Conflict of Interest Statement

The authors declare that the research was conducted in the absence of any commercial or financial relationships that could be construed as a potential conflict of interest.
